# Integrated bulk, single-cell, and spatial transcriptomic analyses prioritize NOTCH1 as a candidate gene associated with neurovascular and immune-related alterations in Parkinson’s disease

**DOI:** 10.3389/fnins.2026.1862571

**Published:** 2026-07-02

**Authors:** Wenkui Li, Jiahao Wei, Changhong Tan, Xi Liu, Lifen Chen

**Affiliations:** 1 Department of Neurology, The Second Affiliated Hospital of Chongqing Medical University, Laboratory Center of Chongqing Medical University, Chongqing, China; 2 Department of Neurology, Chongqing University Three Gorges Hospital, Chongqing, China; 3 School of Medicine, Chongqing University, Chongqing, China

**Keywords:** candidate gene, neurovascular unit, NOTCH1, Parkinson’s disease, substantia nigra

## Abstract

**Introduction:**

Parkinson’s disease (PD) is classically defined by dopaminergic neurodegeneration in the substantia nigra, yet how immune activation is linked to neurovascular dysfunction in the diseased brain remains incompletely understood.

**Methods:**

Here, we integrated bulk substantia nigra microarray expression datasets with single-cell and spatial transcriptomic data to delineate disease-associated neurovascular and immune-related transcriptomic programs in PD.

**Results:**

Across three independent human microarray cohorts, differential expression and weighted gene co-expression network analyses identified PD-associated genes enriched for synaptic processes together with immune, adhesion, and vascular-related pathways. Network topology analysis and machine-learning feature selection prioritized a five-gene candidate panel, among which NOTCH1 showed the most consistent cross-dataset association and external directional support. Importantly, quantitative real-time PCR (qRT-PCR) validation in the substantia nigra of 1-methyl-4-phenyl-1,2,3,6-tetrahydropyridine (MPTP)-induced PD model mice further supported dysregulated Notch1 expression. Single-cell mapping placed NOTCH1 expression within neurovascular and glial cellular contexts, including pericytes and endothelial cells, while CellChat and NicheNet analyses nominated transcriptome-derived ligand-receptor relationships involving NOTCH-related, vascular, inflammatory, extracellular-matrix, and growth-factor-associated programs. Spatial transcriptomics from mouse 6-hydroxydopamine (6-OHDA) substantia nigra sections provided model-based anatomical context for spatial proximity between pericyte and microglial signatures, without establishing direct functional communication. In parallel, exploratory in silico perturbation and docking-based screening generated hypotheses regarding the NOTCH1-associated regulatory context and compounds with predicted docking affinity toward NOTCH1.

**Discussion:**

Collectively, these analyses prioritize NOTCH1 as a reproducible PD-associated candidate gene and suggest that NOTCH1-related signals may be embedded within broader neurovascular, glial, inflammatory, extracellular-matrix, and immune-associated transcriptomic alterations. These findings provide a computational prioritization framework for future experimental validation rather than evidence of a defined NOTCH-driven mechanism.

## Introduction

Parkinson’s disease (PD) is a progressive neurodegenerative disorder and a rapidly growing global health burden (GBD 2016 Parkinson’s Disease Collaborators, 2018; Su et al., 2025; Grotewold and Albin, 2024). It is classically characterized by degeneration of dopaminergic neurons in the substantia nigra with consequent striatal dopamine deficiency, and by the accumulation of Lewy pathology driven by misfolded *α*-synuclein (Bloem et al., 2021; Emin et al., 2022; Spillantini et al., 1997). Despite major advances in human genetics and experimental modelling, the molecular mechanisms that link pathological protein stress to selective neuronal vulnerability remain incompletely understood, particularly in idiopathic disease where no single genetic cause is evident (Ye et al., 2023; Nalls et al., 2019; Kim et al., 2024; Blauwendraat et al., 2020; Morris et al., 2024). Clinically, diagnostic uncertainty is common in early stages when symptoms are subtle and overlap with other neurodegenerative syndromes, and the wide spectrum of motor and non-motor manifestations highlights marked biological and clinical heterogeneity (Tolosa et al., 2021). These challenges underscore the need for molecular signatures that complement clinical assessment and enable mechanism-informed stratification.

Beyond a neuron-centric view, converging evidence indicates that immune dysregulation is a core feature of Parkinson’s disease biology. Genetic studies and integrative disease models support interactions between *α*-synuclein pathology and both innate and adaptive immune responses, including microglial activation and T-cell–related processes (Ye et al., 2023; Tansey et al., 2022; Le Guen et al., 2023; Yan et al., 2021). Human single-cell studies of midbrain tissue have further emphasized this non-neuronal dimension by revealing broad glial activation programs and disease-associated microglial states alongside neuronal changes (Smajić et al., 2022). Together, these observations suggest that mechanistic understanding of Parkinson’s disease requires resolving immune programs in their cellular context and clarifying how immune states interface with other non-neuronal compartments in the diseased brain. In parallel, the neurovascular unit and the blood–brain barrier have emerged as active participants in neurodegenerative pathophysiology. The neurovascular unit comprises endothelial cells, pericytes, astrocytes, neurons, and extracellular matrix, and its integrity is essential for maintaining brain homeostasis (Zlokovic, 2011; Iadecola, 2013). Barrier dysfunction and altered intercellular communication at the vascular–immune interface may facilitate leukocyte trafficking, amplify inflammatory signaling, and contribute to secondary injury cascades (Sweeney et al., 2016; Paul and Elabi, 2022). Notch signaling is particularly relevant in this setting because it mechanistically bridges vascular stability with immune-state regulation (Wang et al., 2014). Notch pathways regulate mural-cell biology and endothelial barrier properties, and experimental evidence in Parkinson’s disease-relevant systems supports a Notch–inflammation interface that can modulate microglial activation programs (Polacheck et al., 2017; Liang et al., 2023). However, how Notch-related immune–neurovascular programs are organized across cell types and communication networks in human Parkinson’s disease, and which mediators link vascular and immune compartments, remains insufficiently characterized.

High-throughput transcriptomics provides a powerful opportunity to address these questions, yet each modality has inherent limitations. Bulk substantia nigra profiling captures dysregulated neuronal and inflammatory programs but is confounded by cell-type heterogeneity and neurodegeneration-driven compositional shifts (Cappelletti et al., 2023; Fiorini et al., 2024), whereas single-cell transcriptomics delineates disease-associated states within defined cell types (Wang Q. et al., 2024) and spatial transcriptomics adds anatomical context by preserving tissue organization and regional patterning (Ma et al., 2025). Therefore, an integrative framework that combines cross-cohort validation, cell-type-resolved mapping, and composition-aware analysis is needed to distinguish reproducible disease-associated signals from bulk expression changes driven mainly by neuronal loss or glial and vascular remodeling.

Here, we applied an integrative multi-modal transcriptomic framework to interrogate substantia nigra pathology in Parkinson’s disease across tissue, cellular, and spatial scales. We analyzed bulk microarray cohorts using differential expression, weighted gene co-expression network analysis (WGCNA), and machine-learning-based feature prioritization to prioritize PD-associated genes. To evaluate reproducibility beyond the merged discovery dataset, we further performed cohort-level validation and fixed-effect and random-effects meta-analyses across independent human bulk datasets. We then mapped candidate genes in human single-cell transcriptomic data and assessed their cell-type distribution and composition-aware disease associations. We reconstructed cell–cell communication networks with CellChat, with ligand–receptor prioritization using NicheNet, to infer potential intercellular signaling relationships rather than to establish experimentally confirmed communication. Spatial transcriptomics (ST) was used to provide 6-OHDA mouse model-based spatial support for neurovascular and immune programs within substantia nigra regions. In addition, scTenifoldKnk was used for exploratory in silico perturbation of NOTCH1 within pericytes, and molecular docking was performed as a hypothesis-generating analysis to prioritize candidate compounds for future validation. Together, these analyses prioritize NOTCH1 as a reproducible PD-associated candidate gene and place NOTCH1-related signals within broader neurovascular, glial, inflammatory, extracellular-matrix, and immune-associated transcriptomic programs. Accordingly, the proposed pericyte- and microglia-associated NOTCH-related patterns are presented as computationally inferred hypotheses requiring future experimental validation, rather than as a defined disease-driving signaling axis.

## Materials and methods

### Data sources and preprocessing

**Figure 1:**
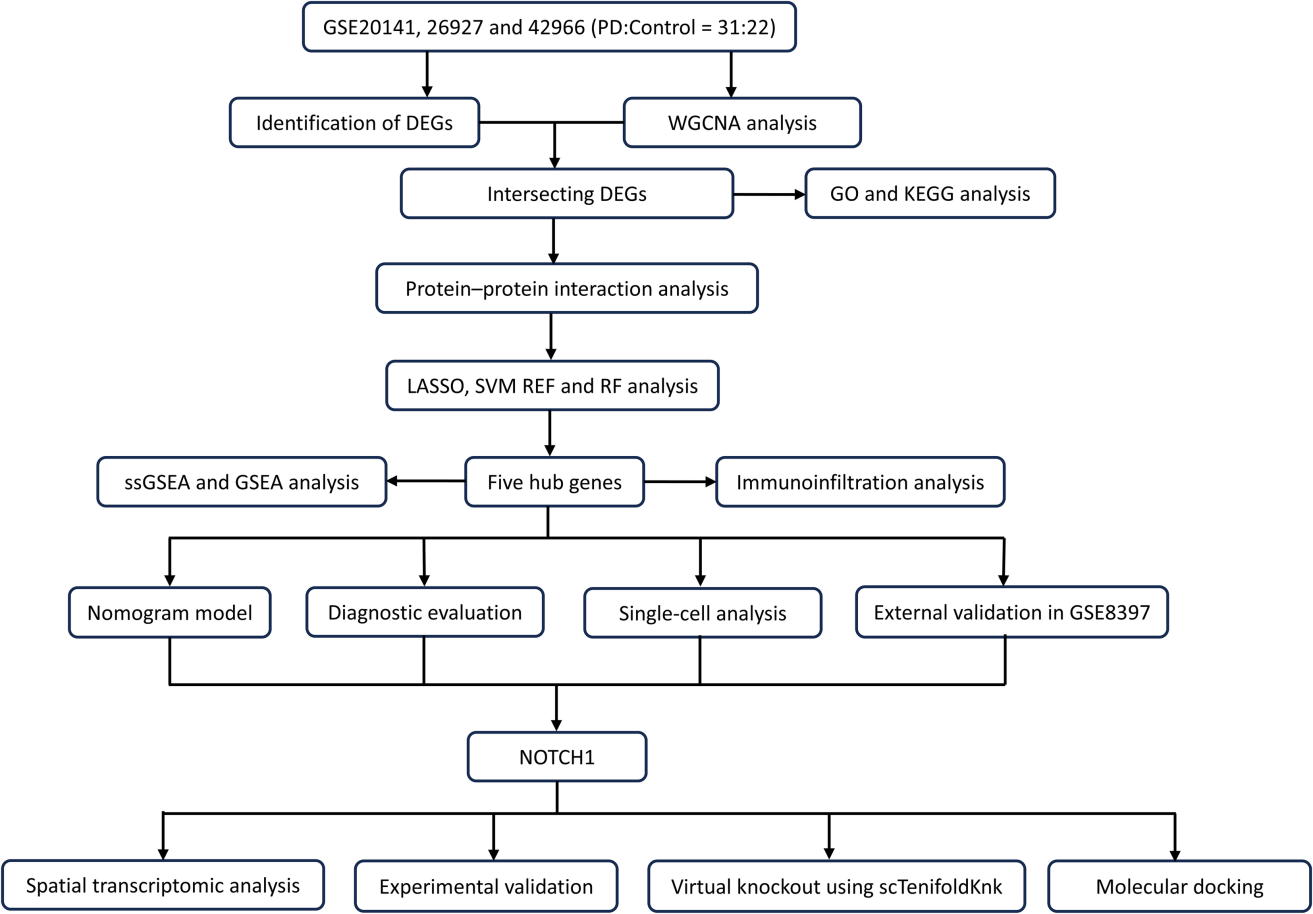
Study workflow and integrative multi-omics analysis pipeline.

An overview of the data preprocessing and analytical workflow used in this study is provided in Figure 1. Raw expression data from three independent human substantia nigra microarray cohorts (GSE20141, GSE26927, GSE42966) comprising 22 control and 31 PD specimens were downloaded from the Gene Expression Omnibus. Probe intensities were mapped to official gene symbols, and probes mapping to the same gene were collapsed by taking the mean. Because the datasets were generated on different platforms, batch effects were assessed via principal component analysis (PCA) and corrected using the ComBat function in the sva package, which applies an empirical Bayes framework to adjust for location and scale differences across batches. After normalization, data from all cohorts were combined for downstream analyses.

Single-cell RNA-seq data from human substantia nigra PD samples (GSE243639), and mouse spatial transcriptomic data (GSE232910) related to PD were downloaded from the GEO. The human substantia nigra single-cell RNA-sequencing (scRNA-seq) dataset included 29 samples, with 15 from the PD group and 14 from the control group. For spatial transcriptomic analyses, five available mouse 6-OHDA substantia nigra sections, including GSM7392318-GSM7392322, were analyzed independently, while striatal sections were excluded to avoid anatomical-region-specific confounding.

### Differential expression analysis

DEGs between PD and control substantia nigra tissues were identified using the limma package. Genes with a false discovery rate (FDR) < 0.05 and |log_2_(fold change)| > 0.585 were considered significant. Volcano plots and heatmaps were generated with ggplot2 to visualize the DEGs.

### Weighted gene co-expression network analysis

To identify gene modules associated with PD, we applied WGCNA to the normalized expression matrix (Langfelder and Horvath, 2008). The soft-thresholding power (*β*) was selected using the scale-free topology criterion. Modules were identified by hierarchical clustering of the topological overlap matrix, followed by dynamic tree cutting. Module eigengenes were correlated with PD status to identify PD-associated modules, and genes from PD-associated modules were intersected with Differentially expressed genes (DEGs) to obtain candidate PD-related genes.

### Feature selection and machine learning

The intersecting set of DEGs and genes from PD-associated modules was subjected to protein–protein interaction (PPI) analysis using STRING to identify hub genes. Candidate feature genes were further refined using three independent feature-selection approaches: (i) least absolute shrinkage and selection operator (LASSO) regression, (ii) support vector machine recursive feature elimination (SVM-RFE) and (iii) random forest. Genes retained by all three methods were considered key feature genes. A feed-forward artificial neural network (ANN) classifier was trained using the neuralnet package, with scaled feature-gene expression as inputs and PD status as the output. The model architecture comprised one hidden layer with five neurons, and the maximum number of iterations was set to 1,000,000. Diagnostic performance was evaluated using confusion matrices and receiver operating characteristic (ROC) analysis, reporting area under the curve (AUC) values with 95% confidence intervals. Univariate and multivariate logistic regression analyses were performed in R to assess independent associations with PD, reporting odds ratios and 95% confidence intervals.

### Cross-dataset meta-analysis of hub genes

To evaluate the reproducibility of hub-gene expression changes across independent substantia nigra cohorts, cross-dataset meta-analysis was performed using GSE20141, GSE26927, GSE42966, and GSE8397. For each candidate hub gene, Hedges’ g was calculated to quantify the standardized expression difference between PD and control samples within each dataset. Pooled effect sizes and 95% confidence intervals were estimated using both fixed-effect and random-effects models. Positive Hedges’ g values indicated higher expression in PD, whereas negative values indicated lower expression in PD.

### Pathway enrichment and immune infiltration analyses

ssGSEA was conducted with the GSVA package using Hallmark gene sets from the MSigDB database. KEGG pathway enrichment analysis was performed using the clusterProfiler package. Correlations between feature gene expression and pathway scores were assessed by Spearman’s rank correlation. Immune cell infiltration was estimated using CIBERSORT to infer the relative proportions of 22 immune cell types in bulk substantia nigra samples, and differences between PD and control groups were evaluated to characterize the immune landscape in PD.

### Single-cell RNA-seq analyses

To reduce potential age-related confounding, three age-outlier donor samples, including GSM7792144, GSM7792131, and GSM7792124, were excluded based on clinical metadata before downstream analysis. The final single-cell RNA-seq cohort included 26 eligible donor samples. Brain single-cell RNA-seq data were processed in Seurat. Cells with <200 or >6,000 detected genes were excluded, and MALAT1 was removed from the gene list. Putative doublets were identified and removed using scDblFinder. After quality control and doublet removal, 109,762 cells were retained for downstream analyses. Batch effects across samples were corrected using Harmony, followed by dimensionality reduction with UMAP (RunUMAP) and graph-based clustering. Cluster marker genes were identified using FindAllMarkers, and major cell types were annotated based on canonical markers. Cell-type proportions were calculated for each sample and compared between PD and control groups. Expression of PD feature genes across cell types and conditions was visualized using dot plots and violin plots.

Single-cell pathway activity was evaluated using GSVA (Subramanian et al., 2005). KEGG gene sets (c2.cp.kegg.symbols.gmt) from MSigDB were used. Within each major cell type, expression profiles were aggregated by group, GSVA scores were computed, and group differences (PD vs. control) were assessed.

### Cell-cell communication analysis

Intercellular communication was inferred using CellChat (Jin et al., 2021). The annotated Seurat object was converted into a CellChat object, and ligand–receptor interactions were inferred using the curated human CellChatDB. To evaluate disease-state-associated communication remodeling, CellChat analyses were performed separately for PD and control samples. Cell types with insufficient cell numbers in either group were excluded, and balanced downsampling was performed within each cell type and disease group to reduce bias caused by unequal cell numbers. Communication probabilities were computed using CellChat with population.size = FALSE to further reduce the influence of group-specific cell abundance differences. To identify global communication patterns, we applied selectK and set nPatterns = 6 for both incoming and outgoing signaling. Global interaction number, interaction strength, pathway-level communication, and selected ligand–receptor pairs were compared between PD and control groups. To interrogate the pathway of interest, we used plotGeneExpression, netVisual heatmap, netVisual chord cell, and netVisual individual to visualize pathway-specific communication (Han et al., 2022). CellChat outputs were used as inferred ligand–receptor communication networks for downstream comparison.

### NicheNet ligand–receptor prioritization

To investigate candidate pericyte-to-microglia ligand-receptor patterns, we applied NicheNet to the integrated scRNA-seq dataset (Browaeys et al., 2020). Pericytes were defined as sender cells and microglia as receiver cells, with PD samples as the condition of interest and controls as the reference. In addition, an all-sender analysis including all non-microglial cell populations was performed to place pericyte-derived ligand signals in the broader multicellular communication context. For ligand–receptor inference, genes expressed in >10% of cells within a cluster were considered expressed. Differentially expressed genes in PD microglia were used as the response gene set. Ligand activity was ranked using the NicheNet ligand-target prior model. The ligand–receptor prior network, top predicted ligand expression across sender cell types, DLL/JAG-NOTCH-related gene expression, and predicted microglial target genes were visualized using heatmaps and dot plots.

### Spatial transcriptomic analysis

ST and single-cell RNA-seq data were integrated to characterize the spatial organization of PD tissue. ST data from GSE232910 were used to analyze five mouse 6-OHDA substantia nigra sections independently, while striatal sections were excluded to avoid anatomical-region-specific confounding. ST data were processed using standard 10x Genomics spatial workflows. To achieve higher-resolution cellular annotation, RCTD was applied to project scRNA-seq-defined cell states onto ST spots (Cable et al., 2022). Because the ST data were mouse-derived whereas the single-cell reference was human-derived, mouse genes were mapped to human orthologs before RCTD analysis. MISTy was used to model neighborhood-level dependencies among inferred cell-type compositions (Tanevski et al., 2022; Khaliq et al., 2024). In addition, spatial ecotypes were defined based on spot-level cell-type compositions, and FGSEA was performed across ecotypes to evaluate regional pathway activity and molecular heterogeneity within the PD tissue microenvironment. For replicate-section analysis, pericyte–microglia overlap was calculated as the minimum of RCTD-inferred pericyte and microglia weights for each spot, whereas the product score was calculated as the product of the two weights, and Spearman correlation analysis was used to quantify spatial association within each section. For lesion-vs-contralateral analysis, the left hemisphere was assigned as contralateral/intact and the right hemisphere as lesioned according to the original GSE232910 annotation; a DA-marker-defined SNc/VTA-focused ROI was defined in the contralateral hemisphere and mirrored to the lesioned hemisphere, with each section analyzed independently.

### Experimental validation in an MPTP mouse model

Male C57BL/6J mice aged 8 weeks were obtained from the Experimental Animal Center of Chongqing Medical University and used for experimental validation. Mice were maintained in a specific pathogen-free animal facility under controlled conditions, with a temperature of 23 °C ± 2 °C, relative humidity of 50% ± 10%, and a 12-h light/12-h dark cycle. Animals were housed in standard cages with no more than five mice per cage and had free access to food and water. Bedding and environmental enrichment were provided. General health, body weight, and behavior were monitored throughout the experiment, and efforts were made to minimize distress and suffering. All animal procedures were approved by the Institutional Animal Care and Use Committee of Chongqing Medical University and were performed in accordance with institutional animal welfare guidelines and ARRIVE recommendations.

For the subacute MPTP-induced Parkinson’s disease model, mice were randomly assigned to the control and MPTP groups, with *n* = 4 mice per group. Mice received intraperitoneal injections of MPTP hydrochloride (30 mg/kg; Beyotime) once daily for five consecutive days. Control mice received an equal volume of saline using the same schedule. Fourteen days after the first injection, mice were deeply anesthetized with 1% sodium pentobarbital (60 mg/kg, intraperitoneally). Adequate anesthesia was confirmed by the absence of pedal withdrawal and corneal reflexes. Under deep anesthesia, mice were euthanized by transcardial perfusion with ice-cold phosphate-buffered saline to remove circulating blood. Death was confirmed by cessation of respiration and heartbeat. The brains were rapidly removed, and substantia nigra tissue was dissected on ice, immediately frozen in liquid nitrogen, and stored at −80 °C until RNA extraction.

Total RNA was extracted from substantia nigra tissue using a TaKaRa total RNA extraction kit according to the manufacturer’s protocol. cDNA was synthesized using PrimeScript RT reagent Kit (TaKaRa). Quantitative real-time PCR (qRT-PCR) was performed with SYBR Green Master Mix (Thermo Fisher) on an ABI StepOnePlus Real-Time PCR System. Primer for Notch1 was designed using Primer-BLAST (forward: 5′-GCCAGCAAGAAGAAGCGGAGAG-3; reverse: 5′-ATTGTCGTCCATCAGAGCACCATC-3). Relative expression was calculated via the 2^−ΔΔCt^ method, with Gapdh as the internal control. Differences between groups were assessed using unpaired *t*-tests.

### Virtual knockout of NOTCH1 using scTenifoldKnk

To explore the putative transcriptomic context of NOTCH1 in pericytes, we performed an in silico knockout using scTenifoldKnk within the pericyte subset (Osorio et al., 2022). scTenifoldKnk infers a gene regulatory network (WT) from single-cell expression data and constructs a corresponding virtual KO network for NOTCH1, then identifies virtually perturbed genes by comparing WT vs. KO networks. Significant perturbed genes were defined at FDR < 0.05. These genes were subsequently subjected to GO (BP/CC/MF) and KEGG enrichment analyses (FDR < 0.05) to characterize NOTCH-related and PD-relevant pathway changes.

### Molecular docking

Candidate compounds identified by drug–gene enrichment analysis were subjected to virtual screening against NOTCH1. The NOTCH1 protein structure was obtained from the Protein Data Bank using PDB ID 9B3N. Protein and ligand structures were prepared by removing crystallographic water molecules, adding hydrogens, and optimizing ligand conformations. Molecular docking was performed using AutoDock Vina, with a grid box centered on the predicted binding site. Binding affinities were recorded in kcal/mol, with more negative values indicating stronger predicted binding affinity. Candidate compounds were compared with reference compounds and negative-control compounds to contextualize the docking results. Top-ranked poses were inspected in PyMOL to visualize binding modes and key interactions. Docking results were interpreted as hypothesis-generating computational predictions rather than experimental evidence of direct NOTCH1 binding.

### Statistical analysis

All analyses were performed in R (version 4.4.0). Continuous variables were compared between PD and control groups using Student’s *t*-test or the Wilcoxon rank-sum test, as appropriate, and categorical variables were compared using the *χ*^2^ test or Fisher’s exact test. Spearman correlation analysis was used to assess associations involving pathway scores, immune infiltration estimates, and spatial cell-type weights. Cross-dataset meta-analysis was performed using Hedges’ g with a fixed-effect and random-effects model, as described above. Multiple testing was controlled using the Benjamini–Hochberg false discovery rate (FDR) where applicable. Unless otherwise specified, all tests were two-sided, and *p* < 0.05 was considered statistically significant.

## Results

### Identification of PD-associated candidate genes from bulk substantia nigra transcriptomes

**Figure 2:**
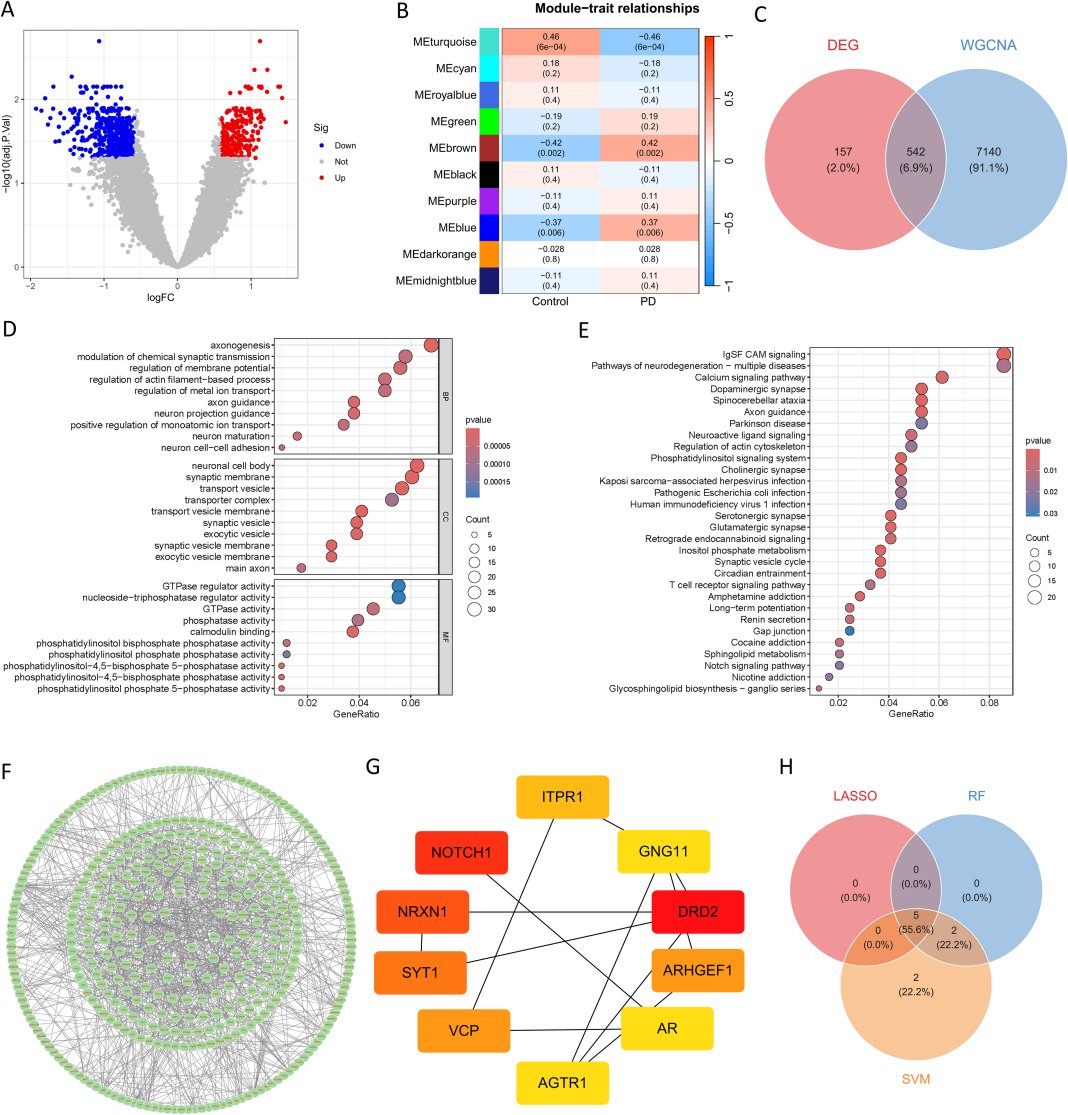
Identification of PD-associated genes and feature-gene selection in the substantia nigra. **(A)** Volcano plot showing differentially expressed genes (DEGs) between PD and control substantia nigra samples. **(B)** Module–trait heatmap showing correlations between WGCNA modules and PD status; each cell reports the correlation coefficient and corresponding *p* value. **(C)** Venn diagram showing 542 overlapping candidate genes obtained by intersecting DEGs with PD-associated WGCNA modules. **(D,E)** Gene Ontology (GO) enrichment **(D)** and Kyoto Encyclopedia of Genes and Genomes (KEGG) pathway enrichment **(E)** analyses of the 542 candidate genes. **(F)** Protein–protein interaction (PPI) network constructed from the candidate genes. **(G)** Top hub genes identified from the PPI network using degree centrality. **(H)** Venn diagram showing five feature genes shared across three feature-selection methods.

To identify molecular features associated with PD in the substantia nigra, we jointly analyzed three microarray cohorts comprising 22 controls and 31 PD samples. After merging the datasets, platform-related batch effects were adjusted using ComBat, which reduced technical inter-cohort variation in PCA visualization (Supplementary Figures S1A,B). Differential expression analysis identified 699 differentially expressed genes (DEGs), including 244 upregulated and 455 downregulated genes (Figure 2A; Supplementary Figure S1C; Supplementary Table S1). Subsequently, we applied WGCNA to construct a scale-free co-expression network and identify modules most strongly associated with PD. Through WGCNA we identified 10 gene modules. The brown and turquoise modules showed the strongest positive and negative correlations with PD status, respectively, whereas the blue module showed a weaker positive correlation (Figure 2B; Supplementary Table S2). Intersecting DEGs with genes from these PD associated modules yielded 542 overlapping genes (Figure 2C; Supplementary Table S3a).

GO and KEGG enrichment analyses indicated that the candidate genes were primarily involved in biological processes related to neuronal projection development, axonogenesis and synaptic signaling, but also pathways involving immune and signaling cascades, including calcium signaling, T cell receptor signaling and Notch signaling (Figures 2D–E). Protein–protein interaction analysis identified 10 hub genes (ARHGEF1, NOTCH1, GNG11, DRD2, VCP, AR, SYT1, ITPR1, AGTR1 and NRXN1) (Figures 2F–G, Supplementary Table S3b). Using these 10 hub genes as input, three feature-selection approaches-LASSO, SVM-RFE, and random forest-converged on five PD feature genes: ARHGEF1, NOTCH1, GNG11, AR, and AGTR1 (Supplementary Figures S1D–I; Supplementary Table S4a). Because this feature-selection workflow was performed within the merged discovery dataset, it was interpreted as candidate prioritization rather than independent validation.

**Figure 3:**
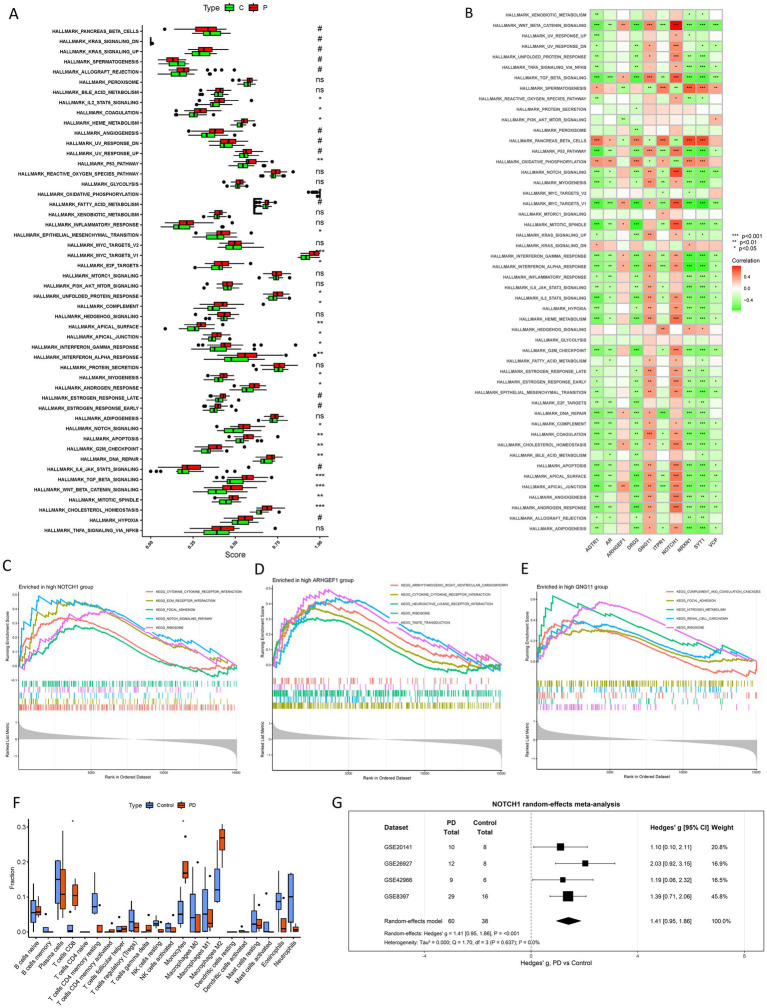
Pathway remodelling and immune landscape associated with PD and feature-gene expression. **(A)** ssGSEA of bulk transcriptomic profiles showing significant enrichment of immune- and stress-related pathways in PD compared with controls. ns, not significant, # = 0.05 ≤ *p* < 0.10, * = *p* < 0.05, ** = *p* < 0.01, *** = *p* < 0.001. **(B)** Correlation heatmap between the 10 PPI-derived hub genes and Hallmark pathway scores. Red indicates positive correlations and green indicates negative correlations. **(C–E)** KEGG pathways enriched in the high-expression groups of NOTCH1 **(C)**, ARHGEF1 **(D)**, and GNG11 **(E)**. Curves show the running enrichment score (ES); positive ES indicates enrichment in the high-expression group, whereas negative ES indicates enrichment in the low-expression group. **(F)** Comparison of inferred immune cell infiltration between PD and control groups. **(G)** Random-effects meta-analysis of NOTCH1 across four independent substantia nigra microarray datasets.

To assess cross-cohort reproducibility, we further evaluated all 10 PPI-derived hub genes across four human substantia nigra datasets, including GSE20141, GSE26927, GSE42966, and GSE8397. Among them, NOTCH1 showed the most consistent positive association with PD. In the random-effects model, the pooled Hedges’ g for NOTCH1 was 1.41 (95% CI: 0.95–1.86; *p* < 0.001), with negligible between-cohort heterogeneity (*I*^2^ = 0.0%) (Figure 3G). Full meta-analysis results for all 10 hub genes are provided in Supplementary Figures S2, S3. These findings support NOTCH1 as a reproducible PD-associated candidate gene emerging from the bulk transcriptomic analysis.

### Pathway remodeling and immune landscape associated with PD feature genes

To determine whether the feature genes were associated with coordinated pathway changes in PD, we performed ssGSEA using Hallmark gene sets. PD samples showed increased activity of multiple immune- and stress-related pathways, including IL2/STAT5 signaling, interferon responses, TGF *β* signaling, apoptosis, p53 signaling, DNA repair and Notch signaling (Figure 3A; Supplementary Table S4b). These results suggest that NOTCH-related changes occurred within a broader immune and stress-response remodeling landscape rather than as an isolated pathway alteration. Correlation analysis indicated that NOTCH1 had particularly strong relationships with pathways reflecting inflammatory activation and signaling remodeling (Figure 3B).

KEGG based GSEA revealed that high expression of ARHGEF1, NOTCH1 and GNG11 enriched cytokine–cytokine receptor interaction, extracellular matrix–receptor interaction, adhesion and inflammation related pathways, together with Notch signaling (Figures 3C–E). Thus, the pathway-level results supported a combined inflammatory, vascular/ECM, and Notch-associated remodeling pattern in PD substantia nigra. Samples with low AR and AGTR1 expression showed reduced enrichment of pathways such as DNA replication, JAK–STAT signaling and Toll-like receptor signaling (Supplementary Figures S4A,B). Immune infiltration analysis further suggested that PD substantia nigra harbors a remodeled immune landscape, characterized by higher inferred proportions of CD8 T cells and monocytes (Figure 3F; Supplementary Figure S4C; Supplementary Table S4c). Because these immune proportions were inferred from bulk transcriptomic data, they were interpreted as supportive evidence of immune remodeling rather than direct quantification of immune-cell abundance.

Together, these analyses indicate that the PD-associated feature genes are embedded within a broader immune, inflammatory, ECM/vascular, and Notch-related remodeling program in the PD substantia nigra.

### Single-cell atlas defines cell-type localization of PD feature genes

**Figure 4:**
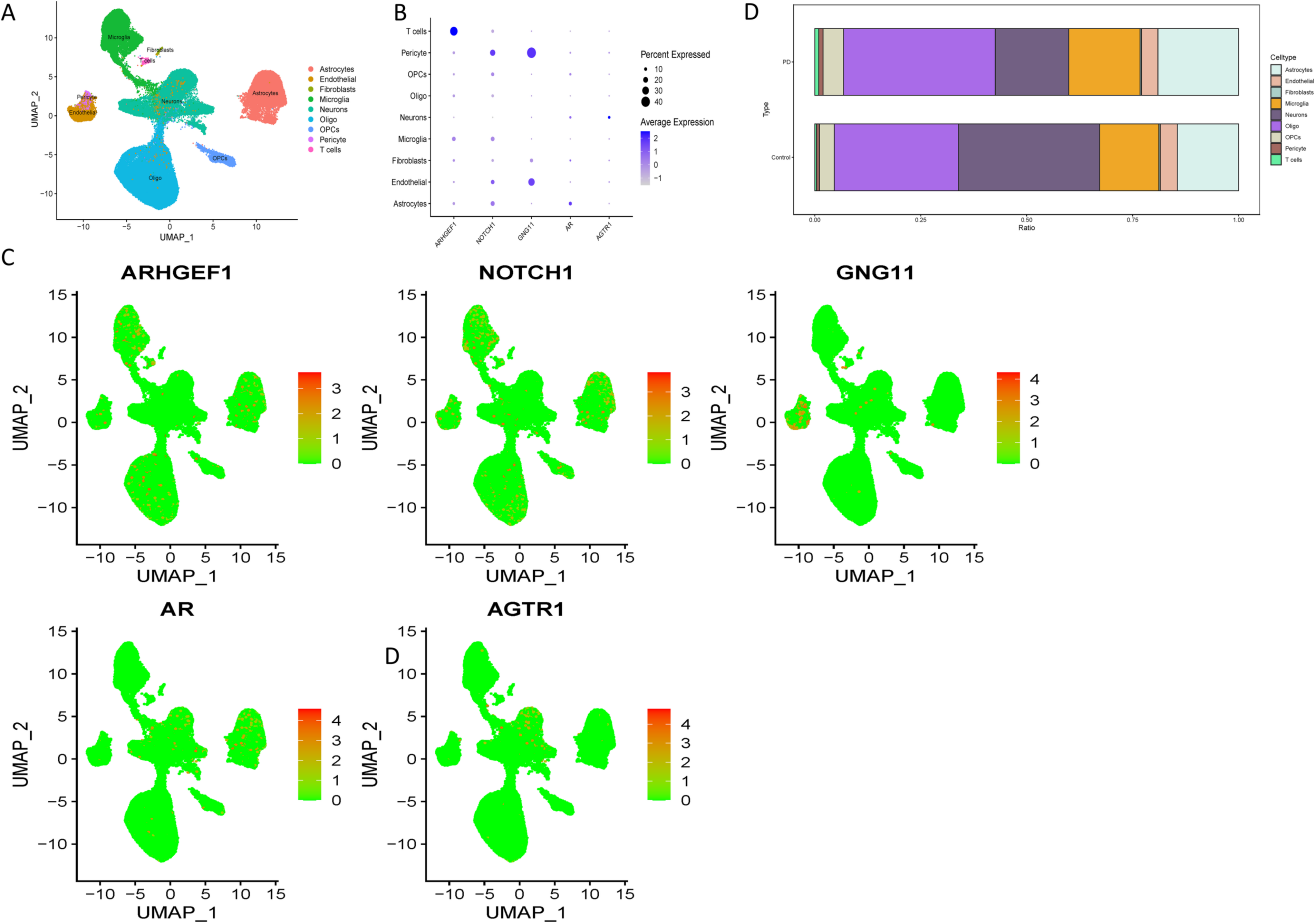
Single-cell atlas of the substantia nigra and cell-type localisation of PD feature genes. **(A)** UMAP visualization of major cell types in substantia nigra scRNA-seq data from PD and control samples. **(B)** Heatmap showing the relative expression (enrichment) of the five feature genes (ARHGEF1, NOTCH1, GNG11, AR, and AGTR1) across substantia nigra cell types. **(C)** UMAP feature plot showing the five feature genes (ARHGEF1, NOTCH1, GNG11, AR, and AGTR1) expression. **(D)** Stacked bar plot showing the relative proportion of each major cell type in PD and control samples.

To define the cellular context of the feature genes, we analyzed single-cell transcriptomic data from PD and control brain tissues. Unsupervised clustering identified 29 clusters assigned to nine major cell populations: neurons, oligodendrocytes, oligodendrocyte progenitor cells (OPCs), astrocytes, microglia, pericytes, endothelial cells, fibroblasts and T cells based on canonical marker expression (Figure 4A; Supplementary Figures S4D,S4E). The fibroblast annotation should be interpreted as a fibroblast-like stromal/perivascular population based on transcriptomic marker expression rather than histological validation of bona fide fibroblasts. Mapping the feature genes across these populations showed broad but non-exclusive cell-type distribution. ARHGEF1 was enriched in T cells; GNG11 showed relatively higher expression in pericytes, endothelial cells, and fibroblasts; AR and AGTR1 were abundant in neurons and astrocytes; and NOTCH1 was detectable in neurovascular-associated populations, particularly pericytes and endothelial cells, while remaining detectable in microglia and astrocytes (Figures 4B,C).

Comparison of cell type composition showed that PD tissues had increased proportions of oligodendrocytes, OPCs, astrocytes, microglia, pericytes and T cells, together with reduced neuronal representation (Figure 4D; Supplementary Table S5). These results suggest that PD substantia nigra is characterized by both cell-type-specific feature-gene expression and broad cellular compositional remodeling. Therefore, we next performed composition-aware analyses to evaluate whether the bulk NOTCH1 signal could be explained by major tissue compositional shifts.

### Composition-aware modeling evaluates the influence of cell-type compositional shifts on bulk hub-gene signals

Because PD substantia nigra is characterized by neuronal loss and relative remodeling of glial and vascular compartments, we next evaluated whether the bulk expression changes of the hub genes could be influenced by cell-type compositional shifts. Cell-type marker scores representing dopaminergic neurons, pan-neurons, microglia, astrocytes, endothelial cells, pericytes, oligodendrocytes, and OPCs were calculated for each bulk substantia nigra sample across the four human datasets (Supplementary Figure S5A; Supplementary Table S6). These marker scores differed between control and PD samples, indicating that bulk transcriptomic profiles were partly shaped by tissue composition.

We therefore re-estimated the PD-associated effects of the 10 hub genes using pooled regression models with increasing compositional adjustment: Model 0 adjusted for dataset only, Model 1 additionally adjusted for global composition PC1, and Model 2 adjusted for selected cell-type marker scores (Supplementary Figure S5B; Supplementary Table S7). NOTCH1 remained positively associated with PD across these models, suggesting that its bulk signal was not fully explained by major cell-type compositional shifts (Supplementary Figure S5C; Supplementary Table S7). In contrast, several neuronal genes, including DRD2, SYT1, ITPR1, NRXN1, and AGTR1, showed stronger dependence on composition-related adjustment. Together, these analyses indicate that NOTCH1 was more stable than several neuronal hub genes after composition-aware adjustment, while confirming that cellular remodeling contributes to the bulk transcriptomic landscape in PD substantia nigra.

### Cell–cell communication analysis suggests PD-associated remodeling of inferred NOTCH-related communication patterns involving neurovascular and glial populations

**Figure 5:**
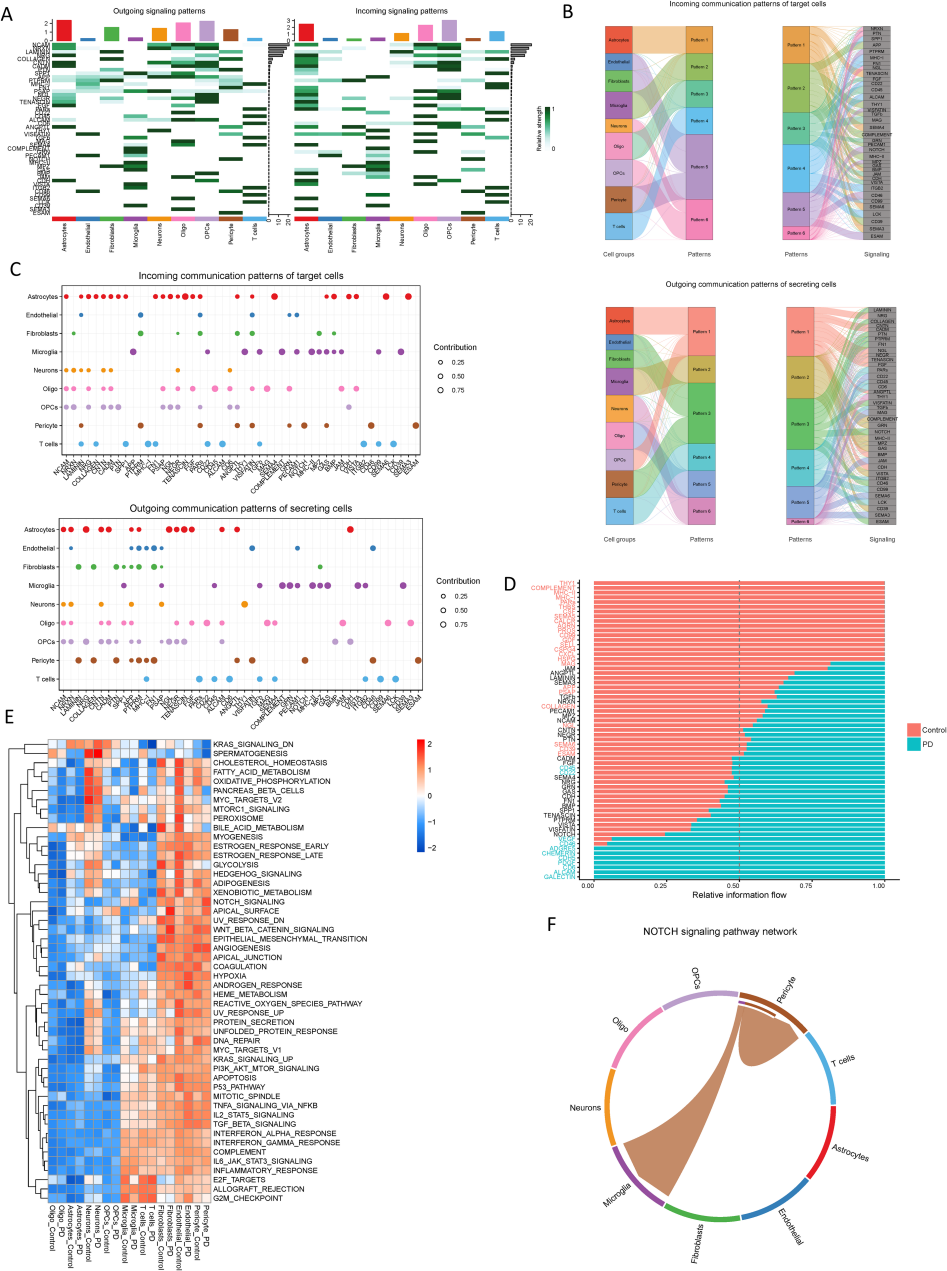
Cell–cell communication inference suggests PD-associated signaling remodeling involving NOTCH-related vascular–immune interactions. **(A)** CellChat-derived outgoing (ligand-enriched) and incoming (receptor-enriched) signaling activities across annotated signaling families. For each pathway, cell-type signaling strength (interaction probability) was row-scaled and visualized; bar plots indicate the total signaling strength across all cell groups. **(B)** Alluvial plot showing inferred incoming and outgoing signaling patterns, linking latent communication patterns with contributing cell groups and signaling pathways. Flow width denotes the contribution of each cell group or pathway to a given latent pattern. **(C)** Dot plot summarizing incoming and outgoing communication patterns across cell groups. **(D)** Signaling pathways ranked by differences in overall information flow between PD and control networks. **(E)** Heatmap of pathway activity differences between PD and controls across cell types based on gene set variation analysis (GSVA); red indicates higher activity and blue indicates lower activity. **(F)** Circle plot of the CellChat-inferred NOTCH communication network. Each arc represents a cell type, and ribbons indicate NOTCH-mediated interactions between cell pairs; the strongest interaction is observed between pericytes and microglia.

Given the expression of NOTCH1 within neurovascular-associated cellular contexts, we next assessed whether transcriptome-derived cell–cell communication patterns involved NOTCH-related signaling among vascular, glial, and immune-related populations. CellChat analysis revealed distinct outgoing and incoming signaling patterns across major cell types (Figure 5A). Vascular-associated populations, including endothelial cells, pericytes, and fibroblasts, were involved in communication patterns containing NOTCH, ESAM, CD46, PTPRM, extracellular matrix-related, and immune-related pathways (Figures 5B,C). RankNet analysis showed that multiple signaling programs differed between control and PD groups, indicating broad communication remodeling rather than an isolated NOTCH-specific change (Figure 5D). Consistently, Hallmark GSVA across cell types showed that NOTCH signaling was embedded within a broader immune, inflammatory, vascular, junctional, hypoxia, interferon, complement, and TNFα/NF-κB remodeling landscape (Figure 5E). Within the inferred NOTCH communication network, pericyte–microglia connections were observed as a prominent component of the NOTCH-related communication structure (Figure 5F).

To directly evaluate disease-state differences, CellChat was then performed separately in control and PD samples after balanced cell-type sampling. Compared with controls, PD samples showed lower total inferred interaction number and overall communication strength, indicating disease-associated communication remodeling rather than global enhancement of cell–cell communication (Supplementary Figures S6A–S6C). Pericyte-to-microglia ligand–receptor analysis identified several predicted interactions, including APP-, CD99-, and NOTCH-related pairs (Supplementary Figure S6D). At the NOTCH-specific ligand–receptor level, a pericyte-to-microglia JAG1-NOTCH2 interaction was detected in PD samples but not in control samples (Supplementary Figure S6D; Supplementary Tables S8–S10). At the pathway-network level, the NOTCH communication network also showed a condition-dependent pattern, with pericyte-related NOTCH links visualized in PD sample (Supplementary Figure S6F). Expression analysis showed that JAG1 was preferentially expressed in pericytes, whereas NOTCH1, NOTCH2, and RBPJ were detectable in microglia; however, canonical downstream targets including HES1, HEY1, and HEY2 showed weak expression in microglia (Supplementary Figures S4F, S6E).

To further evaluate candidate upstream ligand programs involved in the pericyte–microglia communication context, we performed condition-aware NicheNet analysis using microglia as receiver cells. When all non-microglial populations were considered as potential sender cells, NicheNet prioritized NOTCH-related, vascular, extracellular matrix, chemokine, inflammatory, and growth-factor-associated ligands, including DLL1, CXCL12, SPP1, EDN1, TNC, and GAS6 among the top-ranked candidates (Supplementary Figure S4G; Supplementary Table S11). The ligand–receptor prior network showed candidate connectivity among prioritized ligands and receptors, including JAG1-NOTCH1/NOTCH2 interactions (Supplementary Figure S4H). NicheNet-predicted target genes were further examined in microglia, and the top 30 targets showed stronger expression in PD microglia than in control microglia, involving stress-response, inflammatory regulation, immune signaling, and transcriptional remodeling programs (Supplementary Figure S4I).

Together, these results suggest disease-associated remodeling of inferred intercellular communication networks and nominate pericyte- and microglia-associated NOTCH-related candidate communication patterns in PD substantia nigra. Because these analyses are based on transcriptome-derived ligand–receptor inference, these patterns should be interpreted as computationally prioritized candidates rather than validated functional communication or evidence of a defined NOTCH-driven signaling route.

### Feature-gene evaluation supports NOTCH1 prioritization as a PD-associated candidate

**Figure 6:**
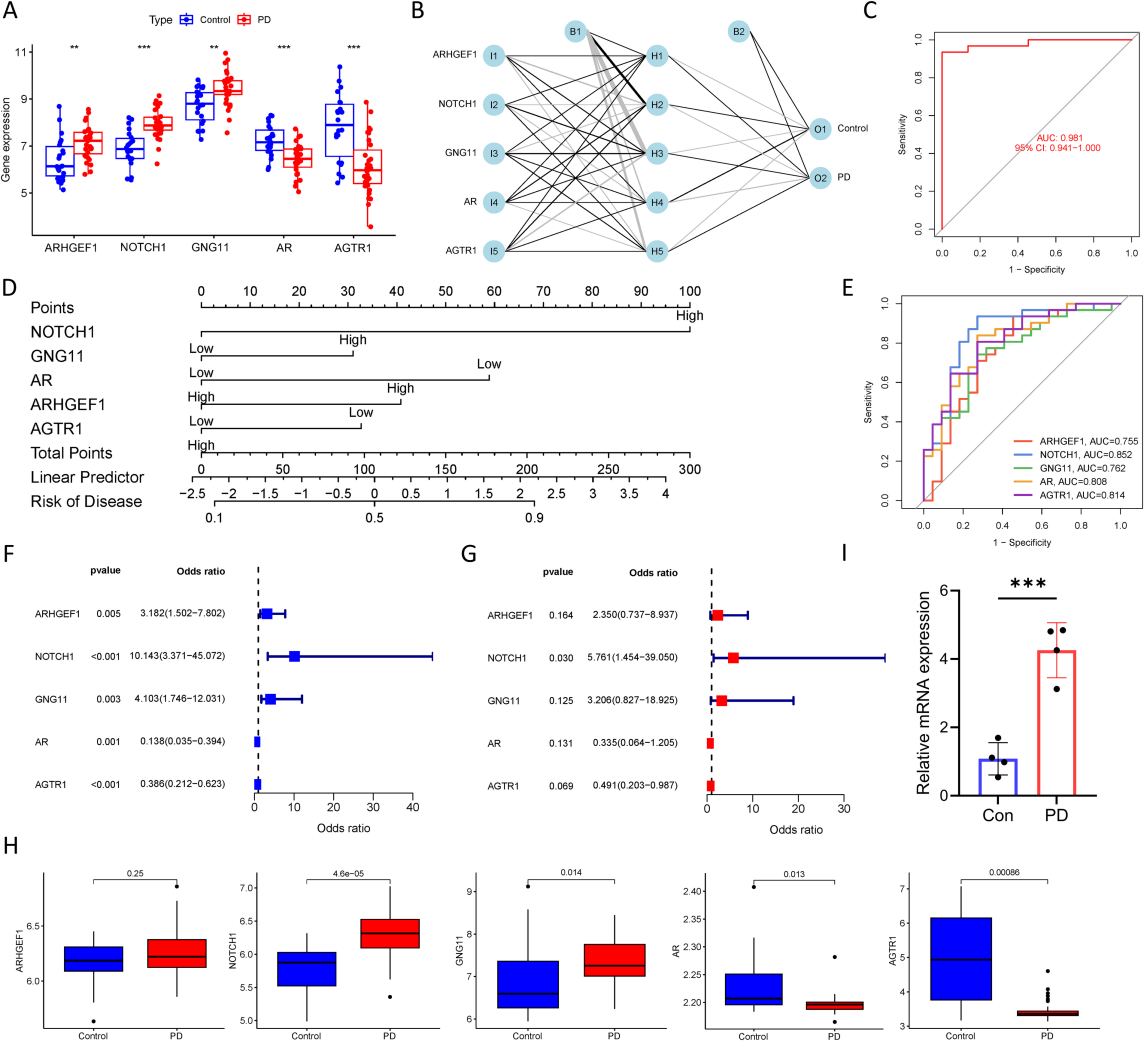
Feature-gene prioritization, external validation, and experimental Notch1 expression validation. **(A)** Expression levels of ARHGEF1, NOTCH1, GNG11, AR, and AGTR1 in PD substantia nigra compared with controls. **(B,C)** Artificial neural network (ANN) classifier built using the five feature genes (ARHGEF1, NOTCH1, GNG11, AR, and AGTR1) as inputs; the model achieved an AUC of 0.981 (95% CI, 0.941–1.000) for distinguishing PD cases from controls. **(D)** Nomogram model of hub genes. The total points, derived from each gene’s expression (low or high), are plotted against the linear predictor and disease risk, with higher points indicating a greater risk of PD. **(E)** Receiver operating characteristic (ROC) curves showing the classification performance of hub genes. **(F,G)** Forest plots of univariate **(F)** and multivariable **(G)** logistic regression analyses for the five feature genes; odds ratios (ORs), 95% confidence intervals (CIs), and *p* values are shown. **(H)** External validation of expression patterns for ARHGEF1, NOTCH1, GNG11, AR, and AGTR1 in an independent substantia nigra cohort (GSE8397). **(I)** The expression of Notch1 was significantly upregulated in substantia nigra of PD mice compared with that in control mice tested by qRT-PCR. Unpaired *t*-test was used for analysis. Data are presented as mean ± SD (*n* = 4). *p < 0.05; **p < 0.01; ***p < 0.001. For boxplots, outliers were displayed according to the standard 1.5 × interquartile range (IQR) rule, and all included samples were retained for statistical testing unless explicitly excluded by predefined quality-control or metadata-based criteria.

Although our study focused on candidate-gene prioritization, we also evaluated the classification performance of the five feature genes in bulk substantia nigra tissue. ARHGEF1, NOTCH1 and GNG11 were significantly upregulated in PD, whereas AR and AGTR1 were downregulated (Figure 6A). A feed-forward artificial neural network trained on the five-gene panel correctly classified all control samples and 93.5% of PD samples (Figures 6B,C). We constructed a nomogram model to visualize PD-associated feature contributions (Figure 6D), which showed consistent classification performance within this dataset. Receiver operating characteristic (ROC) analysis showed that all five genes contributed to PD-associated classification, with area under the curve (AUC) values of 0.755 for ARHGEF1, 0.852 for NOTCH1, 0.762 for GNG11, 0.808 for AR and 0.814 for AGTR1 (Figure 6E). In univariate logistic regression, higher expression of ARHGEF1, NOTCH1 and GNG11 was associated with increased odds of PD, whereas AR and AGTR1 were inversely associated (Figure 6F). In a multivariable model including all five genes, NOTCH1 remained independently associated with PD (OR = 5.76, 95% CI 1.45–39.05; *p* = 0.030) (Figure 6G). External validation in GSE8397 reproduced the direction of expression changes for NOTCH1 and GNG11 (increased in PD) and for AR and AGTR1 (decreased), with ARHGEF1 showing a similar but no significant trend (Figure 6H). Thus, while multiple genes contribute to PD-associated classification in this dataset, NOTCH1 showed consistent association across complementary feature-gene analyses, supporting its prioritization for downstream hypothesis-generating analyses.

### qRT-PCR analysis supports increased Notch1 expression in PD substantia nigra

To validate the transcriptomic findings *in vivo*, we measured Notch1 expression in substantia nigra tissues from 1-methyl-4-phenyl-1,2,3,6-tetrahydropyridine (MPTP)-induced PD mice and saline-treated controls. Quantitative real-time PCR analysis showed that Notch1 mRNA levels were significantly elevated in the PD group (Figure 6I), consistent with the direction of change observed in human transcriptomic data. This result supports disease-associated upregulation of Notch1 in the substantia nigra.

### Spatial transcriptomics provides anatomical context for pericyte and microglial signatures

**Figure 7:**
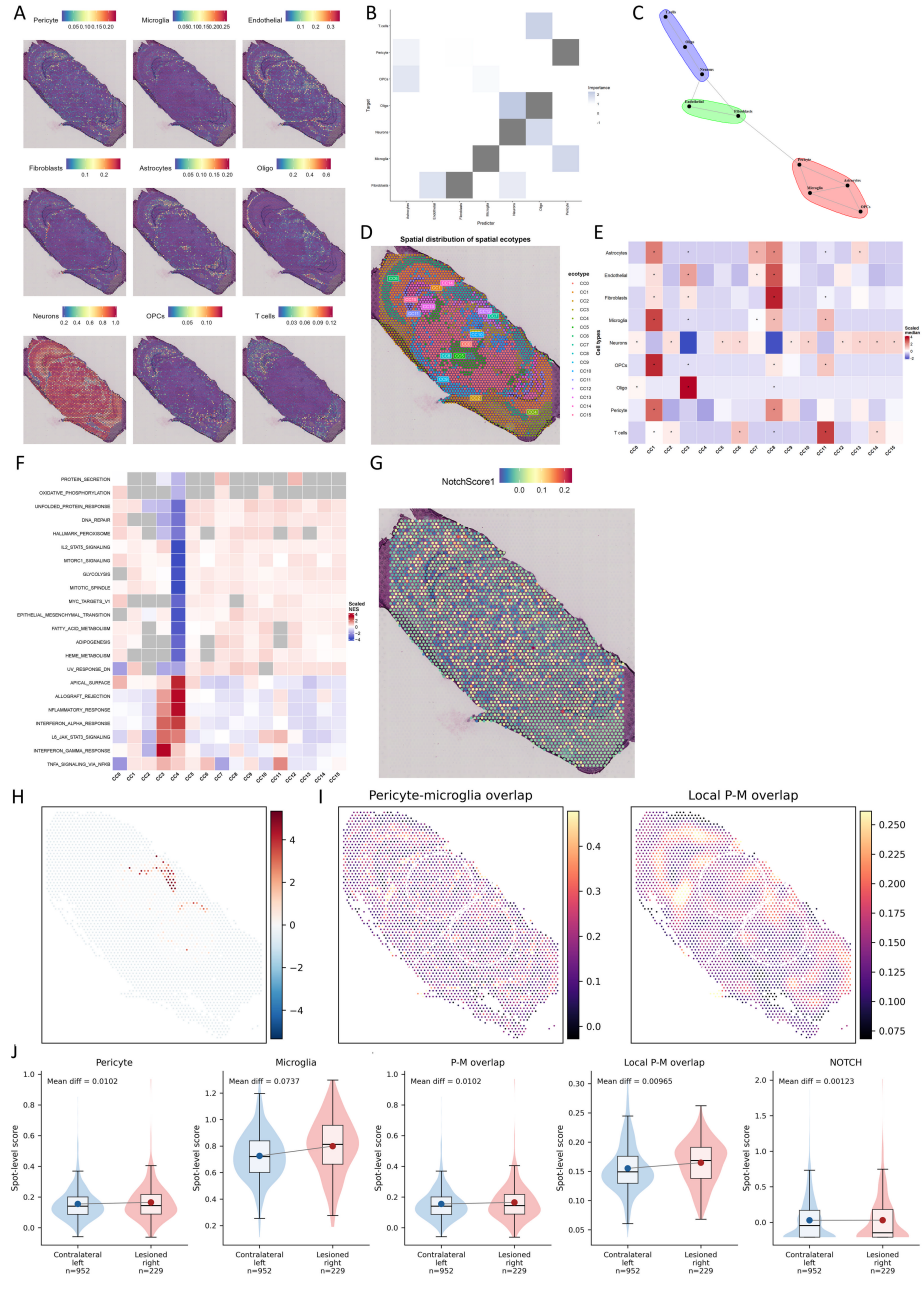
Spatial transcriptomic analysis of mouse unilateral 6-OHDA substantia nigra sections provides anatomical context for pericyte-microglia spatial proximity and NOTCH-associated signals. **(A)** Spatial maps showing the inferred distributions of major cell populations across the representative tissue section. Cell-type abundance was estimated from spatial transcriptomic spots by deconvolution. **(B)** Heatmap of neighborhood-level interactions inferred by MISTy in the paraview. Rows indicate target cell types and columns indicate predictor cell types. Color intensity represents the relative importance of each predictor for modeling the corresponding target in the surrounding spatial neighborhood. **(C)** Community network derived from MISTy paraview analysis, highlighting modular organization of neighborhood-level cell-type interactions. Nodes represent cell populations and edges indicate inferred spatial associations; colored hulls denote interaction communities. **(D)** Spatial distribution of composition-defined spatial ecotypes (CC0–CC15) across the representative tissue section. **(E)** Heatmap showing the scaled median cell-type compositions within each spatial ecotype. Asterisks indicate significant enrichment of a given cell type in an ecotype based on a one-sided Wilcoxon rank-sum test with multiple-testing correction. **(F)** Heatmap of relative normalized enrichment scores (NES) from FGSEA across spatial ecotypes. NES values were scaled across ecotypes for each pathway to highlight relative pathway activity patterns. **(G)** Whole-section spatial distribution of the NOTCH pathway module score (NotchScore1), showing localized variation in NOTCH-associated signaling activity across the representative section. **(H)** Lesion-vs-contralateral spatial reanalysis of the same representative section (GSM7392318). The left hemisphere was assigned as contralateral/intact and the right hemisphere as lesioned according to the original unilateral 6-OHDA dataset annotation. White ellipses indicate the DA-marker-defined SNc/VTA-focused ROI in the contralateral hemisphere and the mirrored ROI in the lesioned hemisphere. **(I)** Spatial distributions of pericyte-microglia overlap and local pericyte-microglia overlap in the same representative section. **(J)** Spot-level distributions of pericyte, microglia, pericyte-microglia overlap, local pericyte-microglia overlap, and NOTCH-associated scores within the SNc/VTA-focused ROI. Blue indicates the contralateral-left ROI and red indicates the lesioned-right ROI. These spot-level patterns are descriptive for the representative section and should be interpreted as anatomical and hypothesis-generating context rather than independent biological replication or evidence of lesion-specific NOTCH-driven spatial rewiring.

To achieve higher-resolution cellular characterization within the ST data, we deconvolved ST spots using a scRNA-seq reference dataset and projected cell identities onto the tissue section with the RCTD algorithm (Cable et al., 2022). The ST data were derived from mouse 6-OHDA substantia nigra sections from GSE232910. This analysis resolved major neural, glial, vascular, stromal, and immune-associated populations, including neurons, oligodendrocytes, OPCs, astrocytes, microglia, endothelial cells, fibroblasts, pericytes, and T cells (Figure 7A). Pericytes and microglia displayed regionally restricted and heterogeneous spatial patterns, suggesting that vascular-associated and inflammatory cell signatures were organized within specific local tissue regions rather than randomly distributed across the section.

To further interrogate higher-order spatial organization, we applied MISTy and spatial ecotype analysis. MISTy revealed structured neighborhood-level dependencies among inferred cell-type compositions, with pericytes, microglia, astrocytes, and OPCs positioned within an interconnected glial–vascular neighborhood framework (Figures 7B,C). Spatial ecotype analysis identified 16 compositionally distinct spot domains (CC0–CC15), with preferential enrichment of selected cell populations, including pericytes and microglia, in defined ecotypes (Figures 7D,E). FGSEA across ecotypes further revealed regional molecular heterogeneity involving inflammatory, immune, metabolic, developmental, and stress-response programs (Figure 7F). In parallel, NOTCH-associated activity showed spatial heterogeneity, with localized regions of relatively elevated signal intensity rather than uniform activation across the tissue (Figure 7G). Because GSE232910 is a unilateral 6-OHDA dataset, we further performed lesion-vs-contralateral reanalysis in the representative section GSM7392318. According to the original dataset annotation, the left hemisphere was assigned as contralateral/intact and the right hemisphere as lesioned. A DA-marker-defined SNc/VTA-focused ROI was defined in the contralateral hemisphere and mirrored to the lesioned hemisphere (Figure 7H). In this representative section, the lesioned-right ROI contained fewer spots than the contralateral-left ROI (229 vs. 952 spots), but showed higher mean spot-level microglial and local pericyte–microglia overlap scores (Figures 7I,J). Because these comparisons were descriptive, based on a single section, and involved unequal ROI sizes, they were interpreted cautiously. NOTCH-associated scores showed only a modest mean difference.

To address section-level reproducibility, we extended the same RCTD-based workflow to five mouse 6-OHDA substantia nigra ST sections from GSE232910, each analyzed independently. Across all five sections, RCTD-inferred pericyte and microglia weights showed positive spatial correlations, and overlap/product scores identified focal regions with simultaneous enrichment of both signatures (Supplementary Figure S7; Supplementary Tables S12, S13a). Lesion-vs-contralateral summary analysis across DA-marker-defined SNc/VTA-focused ROIs showed lesion-side increases in local pericyte–microglia overlap-related scores in GSM7392318 and GSM7392319, but decreased or inconsistent patterns in GSM7392320, GSM7392321, and GSM7392322 (Supplementary Table S13b). Thus, the spatial data support local pericyte–microglia spatial proximity, but not uniform lesion-side NOTCH-associated pericyte–microglia enrichment or lesion-specific NOTCH-driven rewiring.

### Exploratory *in silico* NOTCH1 perturbation nominates pericyte-associated signaling and adhesion-related transcriptomic programs

**Figure 8:**
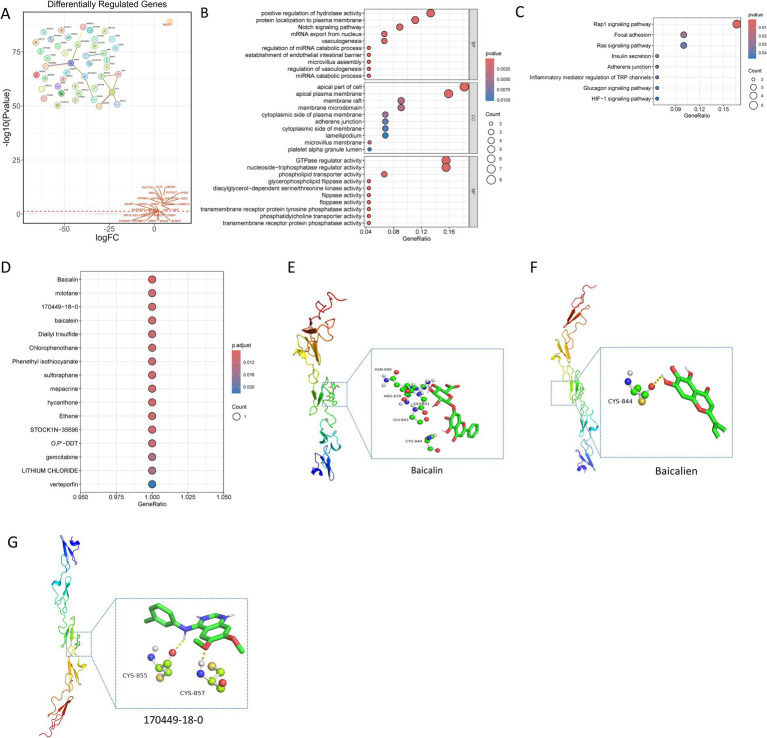
scTenifoldKnk virtual knockout suggests cell-type-associated regulatory changes after NOTCH1 perturbation. **(A)** Virtual knockout (KO) of NOTCH1 in pericytes identifies transcriptional programme perturbations. Gene rank denotes each gene’s position in the scTenifoldKnk-ranked list; QQ plots and STRING-based interconnection networks of significantly perturbed genes are shown. **(B,C)** Gene Ontology (GO) **(B)** and KEGG pathway **(C)** enrichment analyses of pericyte virtual KO–perturbed genes. **(D)** Bubble plot of top enriched compounds from the DSigDB database. Each dot represents a drug signature significantly associated with the five PD feature genes; dot size reflects the number of overlapping genes, and color indicates the adjusted *p* value. **(E–G)** Drug–gene enrichment and molecular docking prioritize compounds with predicted docking affinity toward NOTCH1. Predicted binding poses of baicalin **(E)**, baicalein **(F)**, and 170449-18-0 **(G)** docked to NOTCH1. Complexes were visualized in PyMOL, showing each compound positioned within the predicted binding pocket with key interacting residues highlighted.

Because NOTCH1 was identified as a reproducible PD-associated hub gene and showed enrichment in neurovascular-associated cell populations, we explored its putative regulatory context in pericytes using scTenifoldKnk-based in silico perturbation. Applying an FDR threshold of < 0.05 identified 47 significantly perturbed genes in pericytes (Figure 8A; Supplementary Table S14), including genes linked to vascular or Notch-related programs (HES4, EGFL7, FLT1, VWF, EPAS1, RAPGEF2/4 and GNAQ). Functional enrichment analysis revealed that these perturbed genes were mainly involved in membrane organization, cell junctions, signaling and adhesion, including Rap1 signaling, Ras signaling and focal adhesion pathways. Notably, the virtual perturbation was associated with adhesion- and signaling-related processes rather than generalized transcriptional disturbance (Figures 8B,C). These results provide exploratory computational hypotheses regarding the NOTCH1-associated pericyte transcriptomic context.

### Molecular docking prioritizes compounds with predicted docking affinity toward NOTCH1

Because NOTCH1 was identified as a reproducible PD-associated hub gene through bulk transcriptomic integration, machine-learning prioritization, external validation, and composition-aware analyses, we selected NOTCH1 for exploratory small molecule screening. Drug–gene enrichment identified 16 candidate compounds for docking against NOTCH1 (Figure 8D; Supplementary Table S15). Molecular docking was performed using the NOTCH1 structure. Predicted binding affinities of the candidate compounds are summarized in Supplementary Table S16, and reference compounds and negative-control compounds were included for comparison in Supplementary Table S17a. Detailed docking parameters are provided in Supplementary Table S17b. Virtual screening with AutoDock Vina indicated that baicalin, baicalein, and 170449-18-0 showed the most favorable predicted binding affinities among the candidate compounds (Supplementary Table S16). The reference compounds RO4929097, nirogacestat, and DAPT, together with the negative-control compounds acyclovir, caffeine, and acetaminophen, provided comparative docking controls for contextualizing the candidate compound results (Supplementary Table S17a). Visualization of docking poses suggested plausible hydrogen bonding and hydrophobic interactions within the predicted binding region for baicalin, baicalein, and 170449-18-0 (Figures 8E–G). These docking results should be interpreted as preliminary computational predictions that prioritize compounds for future biochemical target-engagement assays and functional validation, rather than as evidence of direct NOTCH1 binding or therapeutic efficacy.

## Discussion

The primary contribution of this study is the computational prioritization of NOTCH1 as a reproducible PD-associated candidate gene across bulk microarray expression, single-cell, and spatial transcriptomic contexts. Rather than establishing NOTCH signaling as a central disease-driving mechanism, our analyses suggest that NOTCH1-related signals are associated with broader non-neuronal, vascular, glial, inflammatory, extracellular-matrix, and immune-related transcriptomic alterations in Parkinson’s disease substantia nigra datasets. Accordingly, the pericyte- and microglia-associated NOTCH-related patterns described here should be interpreted as hypothesis-generating observations that require future experimental validation. Across complementary analytical layers, NOTCH1 showed reproducible disease-associated expression patterns, cell-type contextualization within neurovascular and glial compartments, and transcriptome-derived associations with inferred ligand-receptor, spatial, and regulatory programs. However, these results do not demonstrate that NOTCH signaling is the dominant or causal mechanism underlying neurovascular or immune remodeling in Parkinson’s disease.

Our findings are broadly consistent with previous studies implicating Notch signaling in PD-related neuroinflammation. Experimental work in MPTP-induced PD models has suggested activation of Notch signaling in microglia, and myeloid-specific disruption of this pathway attenuated microglial inflammatory responses and dopaminergic neurodegeneration (Liang et al., 2023). Building on this framework, our data suggest that NOTCH1 dysregulation in PD may extend beyond immune cells and form part of a broader transcriptional program involving cytokine-cytokine receptor interaction, extracellular matrix remodeling, cell adhesion, and inflammatory signaling. This broader context may be particularly relevant in the substantia nigra, where neuronal vulnerability is closely coupled to vascular and glial dysfunction.

At the cellular level, single-cell analysis placed NOTCH1 expression within neurovascular and glial cellular contexts, including pericyte-associated, endothelial, microglial, and astrocytic populations. Pericytes are multifunctional mural cells involved in blood–brain barrier integrity, angiogenesis, extracellular-matrix organization, and inflammatory regulation, and they may participate in local neurovascular responses under disease conditions (Sweeney et al., 2016; Hattori, 2022; Smyth et al., 2018; Pieper et al., 2014; Guijarro-Muñoz et al., 2014). In this context, CellChat analysis suggested that NOTCH-related signaling was detectable within inferred pericyte-associated communication patterns, while NicheNet nominated transcriptome-derived ligand-receptor relationships involving JAG1 and NOTCH receptors among several vascular, inflammatory, extracellular-matrix, and growth-factor-associated ligand programs. However, these analyses do not demonstrate direct pericyte-to-microglia signaling, nor do they establish that NOTCH signaling is preferentially rewired in PD relative to other disease-associated communication programs. Therefore, the pericyte- and microglia-associated NOTCH-related findings should be interpreted as computationally inferred candidate patterns that require validation using spatial proteomics, multiplex immunostaining, co-culture systems, or cell-type-specific perturbation experiments.

The scTenifoldKnk analysis was retained as an exploratory *in silico* perturbation intended to generate hypotheses regarding the transcriptomic context of NOTCH1 in pericyte-associated cells. Virtual NOTCH1 perturbation identified genes linked to vascular, endothelial, adhesion, and signaling-related programs, including HES4, EGFL7, FLT1, VWF, EPAS1, RAPGEF2/4, and GNAQ. These results suggest that NOTCH1 is associated with pericyte-related structural and signaling programs, but they do not demonstrate a direct regulatory function of NOTCH1 in pericytes or establish downstream causal targets. This interpretation is consistent with prior evidence that Notch signaling can participate in mural-cell/endothelial interactions and vascular stabilization (Tefft et al., 2022), and with reports implicating Notch1-related signaling in PD-associated neural contexts (Wang K. et al., 2024). Accordingly, the scTenifoldKnk results should be interpreted as hypothesis-generating computational evidence that requires validation using cell-type-specific NOTCH1 perturbation, expression assays, and functional experiments in relevant *in vivo* or cell-based systems.

The spatial transcriptomic analysis provided mouse model-based anatomical context for the candidate cellular patterns identified from the single-cell and communication analyses. Because the spatial data were derived from mouse 6-OHDA substantia nigra sections rather than human PD tissue, and because spot-based spatial transcriptomics does not provide single-cell-resolution evidence of physical cell–cell contact, these results should be interpreted cautiously. Across these sections, RCTD-inferred pericyte and microglia weights showed consistently positive spatial correlations, and overlap/product scores identified focal regions with simultaneous enrichment of both cell-type signatures. These findings support reproducible local spatial proximity between pericyte and microglial signatures in the 6-OHDA mouse substantia nigra model, but they do not establish direct ligand-receptor communication, PD-specific spatial rewiring, or a human disease-specific multicellular niche. Thus, the spatial results provide supportive anatomical context for hypothesis generation rather than validation of a NOTCH-driven neurovascular-immune mechanism.

These findings have several potential implications, while remaining hypothesis-generating. First, they highlight the neurovascular unit and immune-related transcriptomic programs as relevant contexts for interpreting PD substantia nigra datasets, complementing the traditional neuron-centered perspective. In particular, the combined observation of NOTCH1 expression in neurovascular-associated cell populations, transcriptome-derived pericyte- and microglia-associated ligand-receptor patterns, and spatial proximity between these two populations suggests that vascular mural cell-related programs may be associated with local inflammatory transcriptomic alterations in PD. However, these observations do not establish direct pericyte-to-microglia signaling or a NOTCH-driven inflammatory mechanism. Second, the reproducible cross-dataset association of NOTCH1 in bulk tissue raises the possibility that NOTCH1 or related transcriptomic programs may serve as indicators of PD-associated substantia nigra alterations, although any clinical or diagnostic biomarker utility will require validation in larger and independent cohorts (Arya et al., 2024). Third, the virtual screening results prioritized several compounds with predicted docking affinity toward NOTCH1, including baicalin and baicalein, which have reported neuroprotective and anti-inflammatory properties and therefore merit experimental testing in PD-relevant systems (Zhu et al., 2019; Rui et al., 2020; Huang et al., 2024). However, the present docking results should be interpreted only as hypothesis-generating computational results for future biochemical, cellular, or animal-model validation, and should not be taken as evidence of direct NOTCH1 target engagement or therapeutic efficacy.

Several limitations should be acknowledged. First, although cross-dataset meta-analysis and composition-aware modeling improved candidate-gene prioritization, the bulk datasets were relatively small, cross-sectional, and derived from microarray platforms. In addition, although ComBat was used to adjust platform-related technical variation, residual biological heterogeneity related to tissue dissection, clinical variability, neuronal loss, and cell-type compositional shifts may remain. Thus, the findings should be interpreted as transcriptomic associations rather than evidence of causal disease mechanisms. Second, the present analyses do not establish NOTCH signaling as the dominant or disease-driving mechanism underlying neurovascular or immune-related alterations in PD. NOTCH1 should instead be viewed as one computationally prioritized candidate signal within broader inflammatory, vascular, extracellular-matrix, glial, stress-response, and immune-associated programs. Third, CellChat and NicheNet infer ligand-receptor relationships from expression patterns and prior knowledge and therefore do not demonstrate direct ligand-mediated communication or functional directionality. Fourth, the spatial analysis was based on mouse 6-OHDA substantia nigra sections and required mouse-to-human ortholog mapping before reference-based RCTD; these spot-based data should be interpreted as model-based spatial association rather than direct validation in human PD tissue. GSM7392318 was used as the representative section because of its relatively complete tissue coverage, whereas the remaining sections varied in completeness and orientation; therefore, multi-section analyses were interpreted as reproducibility assessment of spatial co-patterning rather than evidence of uniform lesion-side remodeling. Finally, scTenifoldKnk and molecular docking are exploratory computational approaches, and their predictions require future validation using cell-type-specific perturbation, spatial proteomics, multiplex immunostaining, biochemical target-engagement assays, and PD-relevant experimental models.

In summary, our multi-layered analyses prioritize NOTCH1 as a reproducible PD-associated candidate gene and place NOTCH1-related signals within broader neurovascular, glial, inflammatory, extracellular-matrix, and immune-associated transcriptomic programs in the substantia nigra. The combined single-cell, communication-inference, and spatial analyses provide cellular and anatomical context for pericyte- and microglia-associated NOTCH-related patterns, but do not establish direct functional communication or a NOTCH-driven disease mechanism. These findings support a computational prioritization framework for future studies aimed at experimentally testing the relevance of NOTCH1-related candidate signals in PD-associated neurovascular and immune-related alterations.

## Conclusion

In this study, we integrated bulk substantia nigra microarray expression datasets with single-cell and spatial transcriptomic data to prioritize candidate genes and cellular contexts associated with Parkinson’s disease. Differential expression, co-expression network analysis, and machine-learning feature selection prioritized a five-gene candidate panel (ARHGEF1, NOTCH1, GNG11, AR, and AGTR1), with NOTCH1 showing reproducible cross-dataset association and external directional support. Single-cell analysis placed NOTCH1 expression within neurovascular and glial cellular contexts, while communication inference nominated transcriptome-derived NOTCH-related ligand-receptor patterns involving pericyte-associated cell populations. Spatial transcriptomics from five mouse 6-OHDA substantia nigra sections provided model-based anatomical context for local proximity between pericyte and microglial signatures. qRT-PCR analysis in an MPTP mouse model supported increased Notch1 expression in substantia nigra tissue, and virtual docking prioritized baicalin, baicalein, and 170449-18-0 as compounds with predicted docking affinity toward NOTCH1. Together, these results prioritize NOTCH1 as a reproducible PD-associated candidate gene and provide hypothesis-generating directions for future experimental validation, rather than evidence of a defined NOTCH-driven neurovascular-immune mechanism.

## Data Availability

The datasets analyzed in this study are publicly available in the NCBI GEO repository. The human substantia nigra bulk transcriptomic datasets include GSE20141, GSE26927, GSE42966, and GSE8397. The single-cell RNA-seq dataset used for cell-type-resolved analysis was GSE243639. The spatial transcriptomic dataset used for mouse 6-OHDA substantia nigra analysis was GSE232910, and the analyzed substantia nigra sections included GSM7392318–GSM7392322. All code and analysis scripts are available from the corresponding author upon reasonable request.

## Glossary

6-OHDA: 6-hydroxydopamine
ANN: Artificial neural network
AUC: Area under the curve
BBB: Blood-brain barrier
CI: Confidence interval
DEGs: Differentially expressed genes
ECM: Extracellular matrix
FDR: False discovery rate
GO: Gene ontology
GSEA: Gene set enrichment analysis
GSVA: Gene set variation analysis
KEGG: Kyoto Encyclopedia of genes and genomes
LASSO: Least absolute shrinkage and selection operator
MISTy: Multiview intercellular spatial modeling framework
MPTP: 1-methyl-4-phenyl-1,2,3,6-tetrahydropyridine
NR: Not reported
NVU: Neurovascular unit
OPCs: Oligodendrocyte progenitor cells
OR: Odds ratio
PD: Parkinson’s disease
PDB: Protein data bank
PPI: Protein–protein interaction
qRT-PCR: Quantitative real-time polymerase chain reaction
RCTD: Robust cell type decomposition
RF: Random forest
ROC: Receiver operating characteristic
scRNA-seq: Single-cell RNA sequencing
SN: Substantia nigra
ssGSEA: Single-sample gene set enrichment analysis
ST: Spatial transcriptomics
SVM-RFE: Support vector machine-recursive feature elimination
WGCNA: Weighted gene co-expression network analysis

## References

[ref1] Arya R. Haque A. Shakya H. Billah M. M. Parvin A. Rahman M. M. . (2024). Parkinson's disease: biomarkers for diagnosis and disease progression. Int. J. Mol. Sci. 25:12379. doi: 10.3390/ijms252212379, 39596444 PMC11594627

[ref2] Blauwendraat C. Nalls M. A. Singleton A. B. (2020). The genetic architecture of Parkinson's disease. Lancet Neurol. 19, 170–178. doi: 10.1016/S1474-4422(19)30287-X, 31521533 PMC8972299

[ref3] Bloem B. R. Okun M. S. Klein C. (2021). Parkinson's disease. Lancet 397, 2284–2303. doi: 10.1016/S0140-6736(21)00218-X, 33848468

[ref4] Browaeys R. Saelens W. Saeys Y. (2020). NicheNet: modeling intercellular communication by linking ligands to target genes. Nat. Methods 17, 159–162. doi: 10.1038/s41592-019-0667-5, 31819264

[ref5] Cable D. M. Murray E. Zou L. S. Goeva A. Macosko E. Z. Chen F. . (2022). Robust decomposition of cell type mixtures in spatial transcriptomics. Nat. Biotechnol. 40, 517–526. doi: 10.1038/s41587-021-00830-w, 33603203 PMC8606190

[ref6] Cappelletti C. Henriksen S. P. Geut H. Rozemuller A. J. M. van de Berg W. D. J. Pihlstrøm L. . (2023). Transcriptomic profiling of Parkinson's disease brains reveals disease stage specific gene expression changes. Acta Neuropathol. 146, 227–244. doi: 10.1007/s00401-023-02597-7, 37347276 PMC10329075

[ref7] Emin D. Zhang Y. P. Lobanova E. Miller A. Li X. Xia Z. . (2022). Small soluble α-synuclein aggregates are the toxic species in Parkinson's disease. Nat. Commun. 13:5512. doi: 10.1038/s41467-022-33252-6, 36127374 PMC9489799

[ref8] Fiorini M. R. Dilliott A. A. Thomas R. A. Farhan S. M. K. (2024). Transcriptomics of human brain tissue in Parkinson's disease: a comparison of bulk and single-cell RNA sequencing. Mol. Neurobiol. 61, 8996–9015. doi: 10.1007/s12035-024-04124-5, 38578357 PMC11496323

[ref9] GBD 2016 Parkinson’s Disease Collaborators (2018). Global, regional, and national burden of Parkinson's disease, 1990-2016: a systematic analysis for the global burden of disease study 2016. Lancet Neurol. 17, 939–953. doi: 10.1016/S1474-4422(18)30295-3, 30287051 PMC6191528

[ref10] Grotewold N. Albin R. L. (2024). Update: descriptive epidemiology of Parkinson disease. Parkinsonism Relat. Disord. 120:106000. doi: 10.1016/j.parkreldis.2024.106000, 38233324 PMC10922566

[ref11] Guijarro-Muñoz I. Compte M. Álvarez-Cienfuegos A. Álvarez-Vallina L. Sanz L. (2014). Lipopolysaccharide activates toll-like receptor 4 (TLR4)-mediated NF-κB signaling pathway and proinflammatory response in human pericytes. J. Biol. Chem. 289, 2457–2468. doi: 10.1074/jbc.M113.521161, 24307174 PMC3900988

[ref12] Han M. Li F. Zhang Y. Dai P. He J. Li Y. . (2022). FOXA2 drives lineage plasticity and KIT pathway activation in neuroendocrine prostate cancer. Cancer Cell 40, 1306–23.e8. doi: 10.1016/j.ccell.2022.10.011, 36332622

[ref13] Hattori Y. (2022). The multiple roles of Pericytes in vascular formation and microglial functions in the brain. Life (Basel). 12:1835. doi: 10.3390/life12111835, 36362989 PMC9699346

[ref14] Huang J. Zhang X. Yang X. Yv Q. Ye F. Chen S. . (2024). Baicalin exerts neuroprotective actions by regulating the Nrf2-NLRP3 axis in toxin-induced models of Parkinson's disease. Chem. Biol. Interact. 387:110820. doi: 10.1016/j.cbi.2023.110820, 38016618

[ref15] Iadecola C. (2013). The pathobiology of vascular dementia. Neuron 80, 844–866. doi: 10.1016/j.neuron.2013.10.008, 24267647 PMC3842016

[ref16] Jin S. Guerrero-Juarez C. F. Zhang L. Chang I. Ramos R. Kuan C. H. . (2021). Inference and analysis of cell-cell communication using CellChat. Nat. Commun. 12:1088. doi: 10.1038/s41467-021-21246-9, 33597522 PMC7889871

[ref17] Khaliq A. M. Rajamohan M. Saeed O. Mansouri K. Adil A. Zhang C. . (2024). Spatial transcriptomic analysis of primary and metastatic pancreatic cancers highlights tumor microenvironmental heterogeneity. Nat. Genet. 56, 2455–2465. doi: 10.1038/s41588-024-01914-4, 39294496

[ref18] Kim J. J. Vitale D. Otani D. V. Lian M. M. Heilbron K. Iwaki H. . (2024). Multi-ancestry genome-wide association meta-analysis of Parkinson's disease. Nat. Genet. 56, 27–36. doi: 10.1038/s41588-023-01584-8, 38155330 PMC10786718

[ref19] Langfelder P. Horvath S. (2008). WGCNA: an R package for weighted correlation network analysis. BMC Bioinformatics. 9:559. doi: 10.1186/1471-2105-9-559, 19114008 PMC2631488

[ref20] Le Guen Y. Luo G. Ambati A. Damotte V. Jansen I. Yu E. . (2023). Multiancestry analysis of the HLA locus in Alzheimer's and Parkinson's diseases uncovers a shared adaptive immune response mediated by HLA-DRB1*04 subtypes. Proc. Natl. Acad. Sci. USA 120:e2302720120. doi: 10.1073/pnas.2302720120, 37643212 PMC10483635

[ref21] Liang S. Q. Li P. H. Hu Y. Y. Zhao J. L. Shao F. Z. Kuang F. . (2023). Myeloid-specific blockade of notch signaling alleviates dopaminergic neurodegeneration in Parkinson's disease by dominantly regulating resident microglia activation through NF-κB signaling. Front. Immunol. 14:1193081. doi: 10.3389/fimmu.2023.1193081, 37680624 PMC10481959

[ref22] Ma M. Paryani F. Jakubiak K. Xia S. Antoku S. Kannan A. . (2025). The spatial landscape of glial pathology and T cell response in Parkinson's disease substantia nigra. Nat. Commun. 16:7146. doi: 10.1038/s41467-025-62478-3, 40759663 PMC12322057

[ref23] Morris H. R. Spillantini M. G. Sue C. M. Williams-Gray C. H. (2024). The pathogenesis of Parkinson's disease. Lancet 403, 293–304. doi: 10.1016/S0140-6736(23)01478-2, 38245249

[ref24] Nalls M. A. Blauwendraat C. Vallerga C. L. Heilbron K. Bandres-Ciga S. Chang D. . (2019). Identification of novel risk loci, causal insights, and heritable risk for Parkinson's disease: a meta-analysis of genome-wide association studies. Lancet Neurol. 18, 1091–1102. doi: 10.1016/S1474-4422(19)30320-5, 31701892 PMC8422160

[ref25] Osorio D. Zhong Y. Li G. Xu Q. Yang Y. Tian Y. . (2022). scTenifoldKnk: an efficient virtual knockout tool for gene function predictions via single-cell gene regulatory network perturbation. Patterns (N Y). 3:100434. doi: 10.1016/j.patter.2022.100434, 35510185 PMC9058914

[ref26] Paul G. Elabi O. F. (2022). Microvascular changes in Parkinson's disease- focus on the neurovascular unit. Front. Aging Neurosci. 14:853372. doi: 10.3389/fnagi.2022.853372, 35360216 PMC8960855

[ref27] Pieper C. Marek J. J. Unterberg M. Schwerdtle T. Galla H. J. (2014). Brain capillary pericytes contribute to the immune defense in response to cytokines or LPS in vitro. Brain Res. 1550, 1–8. doi: 10.1016/j.brainres.2014.01.004, 24418464

[ref28] Polacheck W. J. Kutys M. L. Yang J. Eyckmans J. Wu Y. Vasavada H. . (2017). A non-canonical notch complex regulates adherens junctions and vascular barrier function. Nature 552, 258–262. doi: 10.1038/nature24998, 29160307 PMC5730479

[ref29] Rui W. Li S. Xiao H. Xiao M. Shi J. (2020). Baicalein attenuates Neuroinflammation by inhibiting NLRP3/caspase-1/GSDMD pathway in MPTP induced mice model of Parkinson's disease. Int. J. Neuropsychopharmacol. 23, 762–773. doi: 10.1093/ijnp/pyaa060, 32761175 PMC7745250

[ref30] Smajić S. Prada-Medina C. A. Landoulsi Z. Ghelfi J. Delcambre S. Dietrich C. . (2022). Single-cell sequencing of human midbrain reveals glial activation and a Parkinson-specific neuronal state. Brain 145, 964–978. doi: 10.1093/brain/awab446, 34919646 PMC9050543

[ref31] Smyth L. C. D. Rustenhoven J. Park T. I. Schweder P. Jansson D. Heppner P. A. . (2018). Unique and shared inflammatory profiles of human brain endothelia and pericytes. J. Neuroinflammation 15:138. doi: 10.1186/s12974-018-1167-8, 29751771 PMC5948925

[ref32] Spillantini M. G. Schmidt M. L. Lee V. M. Trojanowski J. Q. Jakes R. Goedert M. (1997). Alpha-synuclein in Lewy bodies. Nature 388, 839–840. doi: 10.1038/42166, 9278044

[ref33] Su D. Cui Y. He C. Yin P. Bai R. Zhu J. . (2025). Projections for prevalence of Parkinson's disease and its driving factors in 195 countries and territories to 2050: modelling study of global burden of disease study 2021. BMJ 388:e080952. doi: 10.1136/bmj-2024-080952, 40044233 PMC11881235

[ref34] Subramanian A. Tamayo P. Mootha V. K. Mukherjee S. Ebert B. L. Gillette M. A. . (2005). Gene set enrichment analysis: a knowledge-based approach for interpreting genome-wide expression profiles. Proc. Natl. Acad. Sci. USA 102, 15545–15550. doi: 10.1073/pnas.0506580102, 16199517 PMC1239896

[ref35] Sweeney M. D. Ayyadurai S. Zlokovic B. V. (2016). Pericytes of the neurovascular unit: key functions and signaling pathways. Nat. Neurosci. 19, 771–783. doi: 10.1038/nn.4288, 27227366 PMC5745011

[ref36] Tanevski J. Flores R. O. R. Gabor A. Schapiro D. Saez-Rodriguez J. (2022). Explainable multiview framework for dissecting spatial relationships from highly multiplexed data. Genome Biol. 23:97. doi: 10.1186/s13059-022-02663-5, 35422018 PMC9011939

[ref37] Tansey M. G. Wallings R. L. Houser M. C. Herrick M. K. Keating C. E. Joers V. (2022). Inflammation and immune dysfunction in Parkinson disease. Nat. Rev. Immunol. 22, 657–673. doi: 10.1038/s41577-022-00684-6, 35246670 PMC8895080

[ref38] Tefft J. B. Bays J. L. Lammers A. Kim S. Eyckmans J. Chen C. S. (2022). Notch1 and Notch3 coordinate for pericyte-induced stabilization of vasculature. Am. J. Physiol. Cell Physiol. 322, C185–C196. doi: 10.1152/ajpcell.00320.2021, 34878922 PMC8791789

[ref39] Tolosa E. Garrido A. Scholz S. W. Poewe W. (2021). Challenges in the diagnosis of Parkinson's disease. Lancet Neurol. 20, 385–397. doi: 10.1016/S1474-4422(21)00030-2, 33894193 PMC8185633

[ref40] Wang K. Liu X. Y. Liu S. F. Wang X. X. Wei Y. H. Zhu J. R. . (2024). Rbm24/Notch1 signaling regulates adult neurogenesis in the subventricular zone and mediates Parkinson-associated olfactory dysfunction. Theranostics 14, 4499–4518. doi: 10.7150/thno.96045, 39113792 PMC11303084

[ref41] Wang Y. Pan L. Moens C. B. Appel B. (2014). Notch3 establishes brain vascular integrity by regulating pericyte number. Development 141, 307–317. doi: 10.1242/dev.096107, 24306108 PMC3879812

[ref42] Wang Q. Wang M. Choi I. Sarrafha L. Liang M. Ho L. . (2024). Molecular profiling of human substantia nigra identifies diverse neuron types associated with vulnerability in Parkinson's disease. Sci. Adv. 10:eadi8287. doi: 10.1126/sciadv.adi8287, 38198537 PMC10780895

[ref43] Yan Z. Yang W. Wei H. Dean M. N. Standaert D. G. Cutter G. R. . (2021). Dysregulation of the adaptive immune system in patients with early-stage Parkinson disease. Neurol Neuroimmunol Neuroinflamm. 8:e1036. doi: 10.1212/NXI.0000000000001036, 34301818 PMC8299515

[ref44] Ye H. Robak L. A. Yu M. Cykowski M. Shulman J. M. (2023). Genetics and pathogenesis of Parkinson's syndrome. Annu. Rev. Pathol. 18, 95–121. doi: 10.1146/annurev-pathmechdis-031521-034145, 36100231 PMC10290758

[ref45] Zhu Q. Zhuang X. Lu J. (2019). Neuroprotective effects of baicalein in animal models of Parkinson's disease: a systematic review of experimental studies. Phytomedicine 55, 302–309. doi: 10.1016/j.phymed.2018.09.215, 30385133

[ref46] Zlokovic B. V. (2011). Neurovascular pathways to neurodegeneration in Alzheimer's disease and other disorders. Nat. Rev. Neurosci. 12, 723–738. doi: 10.1038/nrn3114, 22048062 PMC4036520

