# Supplementary material for: Integrated bulk, single-cell, and spatial transcriptomic analyses prioritize NOTCH1 as a candidate gene associated with neurovascular and immune-related alterations in Parkinson’s disease
Source: Front Neurosci. 2026 Jul 2;20:1862571. doi: 10.3389/fnins.2026.1862571 (PMC13373119; doi:10.3389/fnins.2026.1862571)
Supplement: Supplementary file 1 [file Data_sheet_1.docx]

Fig. S1 Batch correction and feature-gene selection workflow in bulk substantia nigra datasets. (A, B) Assessment of batch-effect correction across cohorts. Boxplots (A) and principal component analysis (PCA) (B) demonstrate reduced inter-cohort variation after adjustment.(C) Heatmap showing expression patterns of the identified differentially expressed genes (DEGs) across PD and control samples.(D, E) LASSO logistic regression applied to the 10 hub genes identifies five genes with non-zero coefficients at the optimal λ determined by 10-fold cross-validation.(F, G) SVM–RFE analysis of the 10 hub genes. Ten-fold cross-validation curves for accuracy and error rate support selection of an optimal subset of nine genes (accuracy = 0.753; error = 0.247). (H, I) Random forest analysis of the 10 hub genes. The out-of-bag (OOB) error stabilizes as the number of trees increases (H), and seven genes with variable-importance scores >1 are identified as the most informative features for distinguishing PD from controls (I).
