# Supplementary material for: Integrated bulk, single-cell, and spatial transcriptomic analyses prioritize NOTCH1 as a candidate gene associated with neurovascular and immune-related alterations in Parkinson’s disease
Source: Front Neurosci. 2026 Jul 2;20:1862571. doi: 10.3389/fnins.2026.1862571 (PMC13373119; doi:10.3389/fnins.2026.1862571)
Supplement: Supplementary file 2 [file Data_sheet_2.docx]

Fig. S2 Random-effects meta-analysis of 10 hub genes across substantia nigra transcriptomic datasets. Random-effects meta-analysis was performed for 10 PPI-derived hub genes across four substantia nigra bulk transcriptomic datasets, including GSE20141, GSE26927, GSE42966, and GSE8397. Forest plots show dataset-specific and pooled effect sizes for ARHGEF1 (A), NOTCH1 (B), GNG11 (C), DRD2 (D), VCP (E), AR (F), SYT1 (G), ITPR1 (H), AGTR1 (I), and NRXN1 (J). Hedges’ g values and 95% confidence intervals are shown for each dataset and the pooled random-effects estimate. Positive values indicate higher expression in PD than in control samples, whereas negative values indicate lower expression in PD. Among these hub genes, NOTCH1 showed consistent upregulation across cohorts with low heterogeneity.
