# Supplementary material for: Integrated bulk, single-cell, and spatial transcriptomic analyses prioritize NOTCH1 as a candidate gene associated with neurovascular and immune-related alterations in Parkinson’s disease
Source: Front Neurosci. 2026 Jul 2;20:1862571. doi: 10.3389/fnins.2026.1862571 (PMC13373119; doi:10.3389/fnins.2026.1862571)
Supplement: Supplementary file 3 [file Data_sheet_3.docx]

Fig. S3 Fixed-effect meta-analysis of 10 hub genes across substantia nigra transcriptomic datasets. Fixed-effect meta-analysis was performed for 10 PPI-derived hub genes across four substantia nigra bulk transcriptomic datasets, including GSE20141, GSE26927, GSE42966, and GSE8397. Forest plots show dataset-specific and pooled effect sizes for ARHGEF1 (A), NOTCH1 (B), GNG11 (C), DRD2 (D), VCP (E), AR (F), SYT1 (G), ITPR1 (H), AGTR1 (I), and NRXN1 (J). Hedges’ g values and 95% confidence intervals are shown for each dataset and the pooled fixed-effect estimate. Positive values indicate higher expression in PD than in control samples, whereas negative values indicate lower expression in PD. NOTCH1 showed consistent upregulation across cohorts.
