# Supplementary material for: Integrated bulk, single-cell, and spatial transcriptomic analyses prioritize NOTCH1 as a candidate gene associated with neurovascular and immune-related alterations in Parkinson’s disease
Source: Front Neurosci. 2026 Jul 2;20:1862571. doi: 10.3389/fnins.2026.1862571 (PMC13373119; doi:10.3389/fnins.2026.1862571)
Supplement: Supplementary file 4 [file Data_sheet_4.docx]

Fig. S4 Pathway enrichment, immune landscape, snRNA-seq annotation, and NicheNet-based characterization of NOTCH-related ligand programs. (A, B) Gene set enrichment analysis showing KEGG pathways enriched in the low-expression groups of AR (A) and AGTR1 (B). Curves represent running enrichment scores. Positive enrichment scores indicate pathway enrichment in the high-expression group, whereas negative enrichment scores indicate enrichment in the low-expression group. (C) Spearman correlation heatmap of 22 inferred immune cell types. (D) UMAP visualization of cell clusters in the substantia nigra snRNA-seq dataset. (E) Dot plot showing expression of representative marker genes used for cell-type annotation. Dot size indicates the percentage of cells expressing each gene, and color indicates average expression. (F) NicheNet ligand–receptor prior interaction heatmap showing predicted interaction potential between candidate ligands and receptors. The heatmap includes NOTCH-related ligand–receptor connectivity, including JAG1–NOTCH1/NOTCH2. (G) Dot plot showing expression of top NicheNet-predicted ligands across major cell populations stratified by disease group. (H) Dot plot showing expression of DLL/JAG–NOTCH-axis genes in major sender cell populations and microglia. (I) Dot plot showing expression of the top 30 NicheNet-predicted target genes in microglia from control and PD groups.
