# Supplementary material for: Integrated bulk, single-cell, and spatial transcriptomic analyses prioritize NOTCH1 as a candidate gene associated with neurovascular and immune-related alterations in Parkinson’s disease
Source: Front Neurosci. 2026 Jul 2;20:1862571. doi: 10.3389/fnins.2026.1862571 (PMC13373119; doi:10.3389/fnins.2026.1862571)
Supplement: Supplementary file 5 [file Data_sheet_5.docx]

Fig. S5 Composition-aware analysis of bulk datasets. A, Cell-type marker scores were calculated for dopaminergic neurons, pan-neurons, microglia, astrocytes, endothelial cells, pericytes, oligodendrocytes, and OPCs in each bulk substantia nigra sample. Boxplots show marker-score differences between control and PD samples across datasets. B, PD-associated effects of the 10 hub genes were estimated using pooled regression models before and after adjustment for dataset, global composition PC1, or selected cell-type marker scores. C, NOTCH1 regression coefficients across composition-aware models showed that NOTCH1 remained positively associated with PD after adjustment for major composition-related signals.
