# Supplementary material for: Integrated bulk, single-cell, and spatial transcriptomic analyses prioritize NOTCH1 as a candidate gene associated with neurovascular and immune-related alterations in Parkinson’s disease
Source: Front Neurosci. 2026 Jul 2;20:1862571. doi: 10.3389/fnins.2026.1862571 (PMC13373119; doi:10.3389/fnins.2026.1862571)
Supplement: Supplementary file 6 [file Data_sheet_6.docx]

Fig. S6 Direct PD-versus-control comparison of inferred cell–cell communication.
A, Total inferred interaction number and interaction strength in control and PD samples after balanced cell-type sampling. B, Circle plots showing overall interaction number networks in control and PD groups. C, Circle plots showing overall interaction strength networks in control and PD groups. D, Bubble plot of predicted pericyte-to-microglia ligand–receptor pairs in control and PD samples. E, DotPlot showing the expression of NOTCH pathway ligands, receptors, and downstream effector genes across cell types and disease groups. F, Inferred NOTCH pathway communication networks in control and PD samples. The network visualization highlights the pericyte–microglia interface as a candidate cellular context for NOTCH-related communication, but does not establish functional ligand–receptor signaling.
