# Supplementary material for: Integrated bulk, single-cell, and spatial transcriptomic analyses prioritize NOTCH1 as a candidate gene associated with neurovascular and immune-related alterations in Parkinson’s disease
Source: Front Neurosci. 2026 Jul 2;20:1862571. doi: 10.3389/fnins.2026.1862571 (PMC13373119; doi:10.3389/fnins.2026.1862571)
Supplement: Supplementary file 7 [file Data_sheet_7.docx]

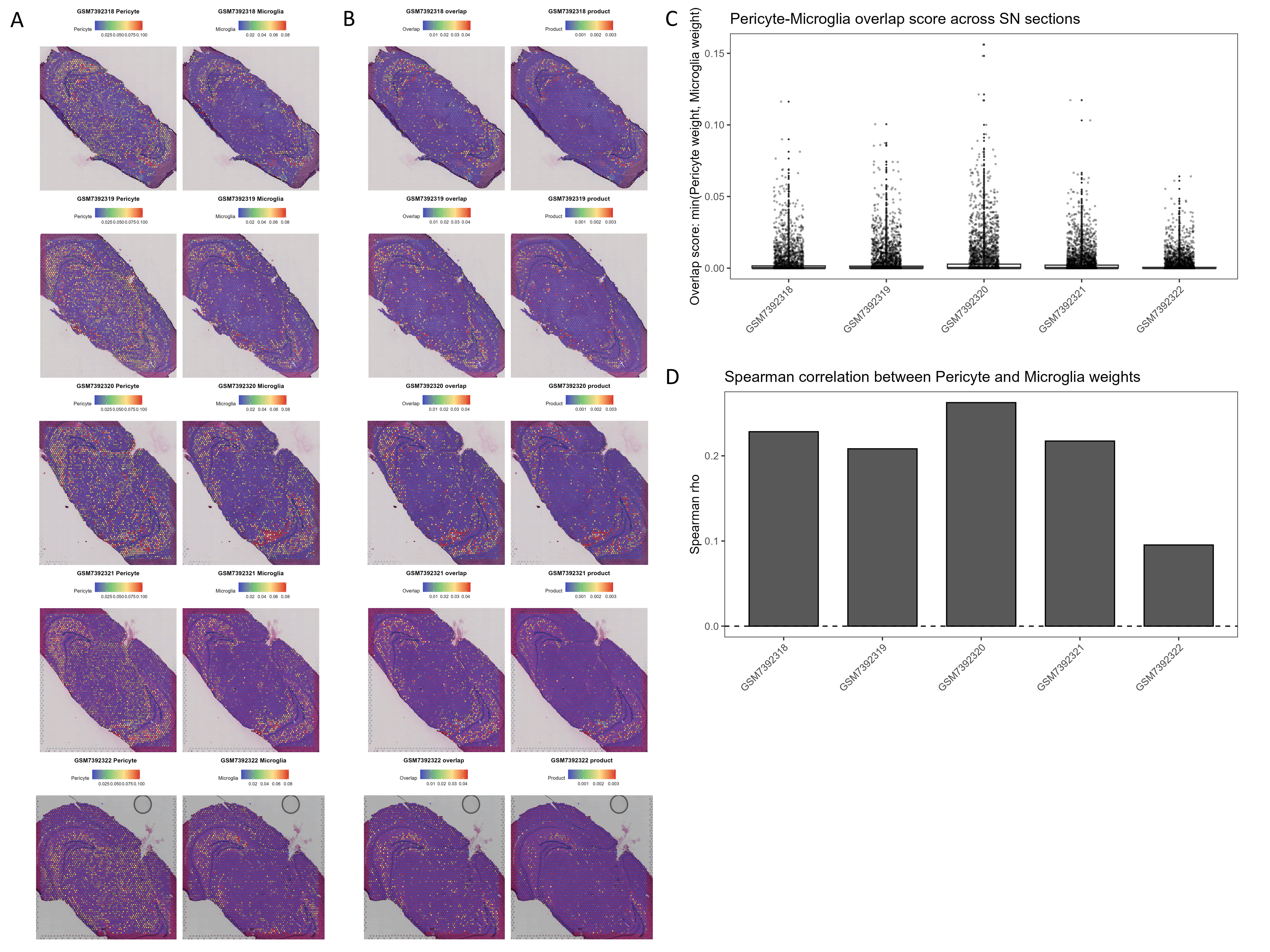


Fig. S7 Replicate spatial transcriptomic analysis of pericyte-microglia spatial association across substantia nigra sections. (A) RCTD-inferred spatial distributions of pericyte and microglia signatures across five substantia nigra spatial transcriptomic sections from GSE232910. (B) Spatial maps of pericyte–microglia overlap and product scores across the five sections. The overlap score was calculated as the minimum of RCTD-inferred pericyte and microglia weights for each spot, whereas the product score was calculated as the product of the two weights. (C) Distribution of pericyte-microglia overlap scores across replicate sections. (D) Spearman correlation coefficients between RCTD-inferred pericyte and microglia weights in each section. Color scales were unified across all sections within each metric to enable direct visual comparison.
